# An environment and comprehensive wellbeing (ECW) conceptual framework: exploring environmental relationships with objective and subjective wellbeing

**DOI:** 10.1007/s11111-026-00518-w

**Published:** 2026-03-28

**Authors:** Laurence Cannings, Craig W Hutton, Kristine Nilsen

**Affiliations:** 1 School of Geography and Environmental Science, University of Southampton, SO17 1BJ, Southampton, UK; 2 Department of Social Statistics and Demography and WorldPop, School of Geography and Environmental Science, University of Southampton, University of Southampton, SO17 1BJ, Southampton, UK

**Keywords:** Environment, Climate, Comprehensive wellbeing, Subjective wellbeing, Objective wellbeing, Framework

## Abstract

Wellbeing and environment are inherently interlinked and, as such, should be supported by a framework that captures multiple relationships between people and ecosystems. A comprehensive wellbeing assessment encompasses broad objective and subjective indicators which account for local community and governance priorities, and acknowledge wellbeing as relational to the time and space it is constructed. While existing frameworks address components of the environment-wellbeing system, they often do not incorporate comprehensive wellbeing within an environmental context. The Environment and Comprehensive Wellbeing framework addresses this gap, supporting research on the impacts of climatic hazards, landscape characteristics, and environmental policy interventions upon wellbeing in low- and middle-income countries. The framework is novel in its exploration of how objective and subjective wellbeing interact, and how the relational context, including environmental conditions over various timescales, impact these relationships. Despite being primarily designed to explore local wellbeing, the framework contains sufficient breadth to facilitate multiscale research. The framework is applied to a mixed-method example in Volta Delta, Ghana. The prominence of subsistence agriculture within the environmentally vulnerable delta accentuates the interconnectivity between livelihoods, wellbeing and environment. Applying this novel framework provides a platform for a deeper understanding of the findings. For example, a context-specific dichotomy between low objective and high subjective wellbeing was illustrated in drought-impacted agricultural areas. Research deploying an exclusively objective approach may have omitted information on how collective community norms could promote sustainable activities, while a subjective approach may have overlooked requirements for more-immediate material support, highlighting the synergistic benefit of encompassing subjective and objective wellbeing.

**Supplementary Information:**

The online version contains supplementary material available at 10.1007/s11111-026-00518-w.

## Introduction

Wellbeing and environment are intrinsically interlinked; therefore, understanding the multidirectional relationships between human needs and supporting ecosystems is crucial for sustainable development. ‘Environment’ can be interpreted as an external wellbeing determinant or an intrinsic constituent (Schleicher et al., 2018). These different concepts illustrate the importance of using a flexible conceptual framework to incorporate different ways of thinking within research.

Wellbeing is defined as a “favourable state of life desirable for every human…in the world at all times” (Böhnke & Kohler, 2010; p.5). However, the conceptualisation is debated across disciplines (Dodge et al., 2012). For example, objective wellbeing (OWB), which incorporates universal, measurable components relating to quality of life, such as income (Western & Tomaszewski, 2016), is common within economics and development studies (Eid & Larsen, 2008). In contrast, subjective wellbeing (SWB), which captures individuals’ internal assessments and affective reactions (Stone & Mackie, 2013), features prominently within psychology (Cooke et al., 2016).

There is no consensus on whether OWB and SWB align or oppose (Böhnke & Kohler, 2010; Oswald & Wu, 2009). For example, studies show objective financial wellbeing to enhance SWB by increasing autonomy (Osei-Tutu et al., 2020), aiding shock recovery (Markussen et al., 2018; Tay et al., 2018), and helping create social relationships (Dzokoto et al., 2019). Yet, opposing OWB and SWB features in the eminent Easterlin (1974) Paradox, where economic growth increases short-term happiness, yet lowers long-term returns by creating unreachable, elevated aspirations, and altering individuals’ relative social positions (Clark et al., 2008; White, 2016). Another potential reason for the disconnect relates to Sen’s (1999) capabilities approach, where money represents ‘potential’ wellbeing, yet skills, rights and institutional support are required to translate 'potential' into ‘realised’ SWB (Gasper, 2007). Due to the different relationships within the literature, wellbeing frameworks should illustrate the capacity for OWB and SWB to be independent and/or interconnected, and operate in tandem and/or opposition.

Wellbeing research has increased due to its growing importance as a policy outcome (Osei-Tutu et al., 2020). Wellbeing is a vital prerequisite, and outcome of, long-term sustainable development within low-and middle-income countries (LMICs) (Helne & Hirvilammi, 2015), illustrated by all UN Sustainable Development Goals (SDGs) explicitly (i.e., SDG3^1^) or implicitly (i.e., SDG2^2^) referring to wellbeing. Achieving wellbeing governs individuals’ behaviour and actions, and therefore it is a key area to address when tackling sustainability issues (White, 2016). Embedding wellbeing within sustainability programmes can also mitigate negative trade-offs which could arise if efforts to reduce environmental vulnerability do not account for local priorities and practices (Angnuureng et al., 2023). Furthermore, wellbeing is “centred in the person”, which alongside the absence of an agreed-upon definition, allows wellbeing to be discussed freely within communities to address challenges in the local context (Atkinson, 2013). The complexity of wellbeing facilitates a holistic outlook, offering opportunities for multidisciplinary, collaborative research targeting different elements of individuals’ and communities’ lives (Agarwala et al., 2014).

Exploring the intersection between environment and wellbeing also contributes to the field of social demography (Piringer et al., 2024), as environmental and climatic shocks have well-documented implications for demographic processes (Thiede et al., 2022), household dynamics (Awiti, 2022), health outcomes (Campbell-Lendrum et al., 2023), and migration patterns (Di Falco et al., 2024; Mueller et al., 2020). Changes in wellbeing may function both as responses to, and drivers of, demographic outcomes. For example, reduced employment opportunities (OWB) following prolonged droughts may promote mass emigration (Appiah, 2025), which can subsequently contribute to family separation, reduced community cohesion (Husile, 2025), and lower SWB due to feelings of abandonment (van der Geest, 2011).

This paper introduces a new conceptual framework to structure and conceptualise the complex environment-wellbeing system (Vaznonienė, 2014). Nunan (2015) outlines two framework approaches when studying poverty^3^ and environment; livelihood and wellbeing. Both approaches seek to improve understanding of human–environment relationships and identify interventions for enhancing wellbeing, but they differ in methodology and how they conceptualise wellbeing. Livelihood approaches, such as the Sustainable Livelihoods Framework (Scoones, 1998), identify general trends and groupings related to resource access and livelihood strategies, often through quantitative methods. Wellbeing approaches, such as the Wellbeing in Developing Countries framework (Gough & McGregor, 2007), understand actors’ emotions and perspectives, commonly through mixed methods. However, the two approaches overlap, as livelihood strategies may influence how environmental conditions impact wellbeing, or how wellbeing outcomes are conceptualised. The novel conceptual framework introduced in this paper draws on both approaches to facilitate deeper understandings of how environmental characteristics may shape various OWB and SWB elements.

To our knowledge, no framework captures the different elements of comprehensive wellbeing explicitly within the context of environmental risk and vulnerability; encompassing the impacts of both climate hazards and landscape characteristics (Walsham, 2020). The comprehensive approach includes: (i) broad selection of indicators, (ii) acknowledges the relational context by reflecting local priorities and contextual factors within time and space, (iii) objective *and* subjective elements (White, 2016). Additionally, many existing environment-wellbeing frameworks possess limitations, such as incorporating limited feedback effects and interpreting wellbeing as a homogenous outcome. By combining elements of existing frameworks, this paper introduces the ECW framework (Environment and Comprehensive Wellbeing). The framework primarily supports wellbeing measurement and research on environment-wellbeing relationships in rural LMICs; yet can be flexibly applied in diverse contexts.

The ECW framework is novel in encompassing elements from livelihood and wellbeing approaches, supporting research on how OWB and SWB interact, and how multiscale environmental conditions and relational contexts impact OWB and SWB differently. By incorporating multidisciplinary concepts, the framework addresses concerns that “by prioritising [OWB], other dimensions…can be ignored/damaged” (Copestake, 2008; p.591). For example, purely objective approaches could omit information on how communities’ collective norms, a foundation of SWB, could be used to harness knowledge sharing and cooperative action to support environmental policies (Bouma et al., 2008). In contrast, exclusively subjective approaches may result in the “grumbling rich man” being prioritised over the “contented peasant” (Sen, 1983; p.160), where defining objectively poor households as content can romanticise financial poverty (Kay & Jost, 2003) and discourage material investments (Davis, 2014). Therefore, sustainability policy should incorporate OWB *and* SWB to improve communities’ comprehensive wellbeing and overcome limitations of singular approaches. See Appendix (1) for further discussions on OWB and SWB within policy. This paper firstly outlines examples of environment-wellbeing relationships, before introducing the ECW framework and supporting evidence from a coastal Ghana case study.

### Environment & wellbeing

Environmental conditions (i.e., climate hazards & landscape characteristics) can be wellbeing determinants *and* constituents. Firstly, environmental conditions can impact OWB. For example, due to the interconnectivity between ecosystem services (ES), food security, and livelihoods within agricultural communities, periods of drought can reduce productivity, trade incomes, and health (Cazcarro et al., 2018). Climate hazards may also affect other OWB elements. For instance, education may be hampered if flooding damages school infrastructure (Addo et al. 2018a), or if subsistence labour is prioritised over schooling during periods of scarcity (Korboe et al., 2011).

Environmental conditions may also influence SWB. For example, Sekulova and van den Bergh (2016) found experiences of flooding, and fears of future flooding to cause long-term SWB reductions. Uncertain future conditions are associated with anxiety (Kidner, 2007; Stokols et al., 2009), while displacement or degradation can also lower SWB if individuals are emotionally connected to the land (Albrecht et al., 2007). Furthermore, many studies highlight the importance of fulfilling intergenerational farmer identities and cultural obligations to provide for the community in agricultural landscapes (Brown et al., 2021; Schaafsma & Gross-Camp, 2021). Therefore, climate-induced reductions in productivity could lower SWB if it prevents individuals from fulfilling such social norms (Anguelovski & Alier, 2014). Additionally, environmental vulnerability is heterogeneous across scales, creating winners and losers (Adger et al., 2002). Relative comparability to others, and one’s previous position, are pivotal SWB controls (Ravallion, 2014). Therefore, increased inequality following hazards, or reduced productivity compared to the past, could lower SWB.

'Environment' can also be interpreted as a wellbeing constituent, with Jax et al. (2018) stating ‘caring for nature’ is a universal psychological need which contributes towards SWB. Natural environments and cultural ES can also generate place attachment and shape identities, representing a source, rather than a means to, wellbeing (Knippenberg et al., 2018). The constituent perspective is also reflected in many traditional worldviews, such as ‘Ubuntu’ in Africa which promotes compassion between human-natural-spiritual elements, thereby aligning environmental wellbeing with communities’ collective wellbeing (Mawere, 2012; Shoko, 2016).

### Existing frameworks

Drawing on Schleicher et al.’s (2018) review, this section briefly introduces several prominent environment-wellbeing frameworks (Agarwala et al., 2014; Fisher et al., 2013). A critical evaluation of the frameworks and illustrative diagrams are presented in Appendices (2–3). As mentioned, certain frameworks interpret ‘Environment’ as a wellbeing determinant; for example, Environmental Endowments and Entitlements (EEE) (Leach et al., 1999) and Driver–Pressure–State–Welfare–Response (DPSWR) (Cooper, 2013). Other frameworks primarily position ‘Environment’ as a determinant, yet can be adapted to incorporate the constituent perspective, including the Millennium Ecosystem Assessment (MEA, 2005), Sustainable Livelihoods Framework (SLF) (Scoones, 1998), Vulnerability framework (Scott, 2006), and IPCC (2024) risk framework. In contrast, two notable frameworks principally interpret ‘Environment’ as a wellbeing constituent, yet also retain flexibility to examine determinant relationships: the Wellbeing in Developing Countries (WeD) (Gough & McGregor, 2007) and the Economics of Ecosystems and Biodiversity (TEEB, 2022) frameworks.

While acknowledging limitations in these frameworks, many elements are incorporated into the novel ECW framework. For example, it incorporates SLF capitals to support quantification of environment–wellbeing relationships, adopts WeD’s ‘relational context’ to recognise how OWB and SWB are constructed across time and space, and applies the IPCC risk framework to interpret OWB and SWB not only as outcomes, but as means to further wellbeing through the influence on adaptive capacity and sensitivity. Further details on the elements incorporated within the ECW framework, and the limitations it addresses, are provided in Appendix (2).

## ECW framework

**Fig. 1:**
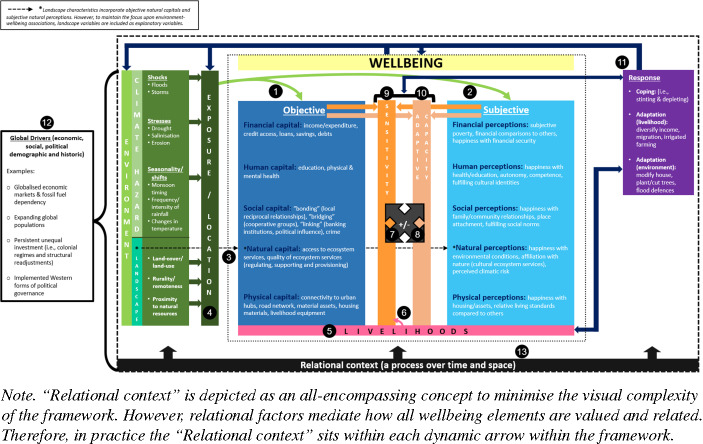
The Environment and Comprehensive Wellbeing (ECW) conceptual framework. The numbered components are discussed in the Volta Delta case study. A version of the ECW framework without the numbering is available in Appendix (4)

The novel ECW framework (Fig. 1) supports research on context-specific environment-wellbeing relationships (Schleicher et al., 2018), and overcomes existing frameworks’ limitations (Appendix 2). These limitations include; focusing on one scale, incorporating limited wellbeing elements, and ignoring underlying governance structures.

While existing frameworks address components of the environment-wellbeing system, they do not incorporate all elements of comprehensive wellbeing within an environmental context. For example, comprehensive approaches consider both objective and subjective elements, yet SLF focuses on livelihood capital access without acknowledging how capitals may influence SWB differently across groups and spaces. Furthermore, the ECW framework acknowledges wellbeing as an outcome and a means to further wellbeing by controlling adaptive capacity and sensitivity, unlike SLF which only incorporates capitals as means to poverty alleviation and wellbeing (Fisher et al., 2014). Similarly, MEA studies often rely on the narrow five wellbeing components defined by Petesch & Narayan, (2002); financial/material, health, security, social relations and freedom of choice. While some elements could be captured through OWB and SWB approaches (Duku et al. 2022a), there remains limited scope to acknowledge context-specific understandings of a 'good life'; another key element of comprehensive wellbeing (White, 2016). This challenge is compounded by the macroscale indicators often used in research using frameworks such as MEA, TEEB and DPSWR (Santos-Martín et al., 2013), which arguably restrict the capacity to explore social differentiation and contextual nuances (Fisher et al., 2014; Nunan, 2015). Nevertheless, some studies have addressed this limitation by adapting the MEA framework to incorporate different groups’ value systems, which subsequently influence how wellbeing is impacted by environmental characteristics (Sangha et al., 2011). While existing frameworks have drawn on the prominent approaches in Appendix (2), such as the Ecosystem Services and Poverty Alleviation (ESPA) framework which incorporates elements from MEA, EEE, and SLF (Fisher et al., 2014), questions remain as to whether it facilitates a comprehensive approach. For example, although ESPA captures actors’ subjective ES preferences for achieving OWB, it does not conceptualise SWB as an outcome in itself.

The ECW framework addresses these potential limitations by illustrating the varying links between climatic hazards, landscape characteristics, and OWB and SWB. The framework incorporates eight components; *Environment*, *Exposure*, *Wellbeing*, *Adaptive capacity & Sensitivity*, *Livelihoods*, *Response*, *Global drivers* and *Relational context* (Fig. 1). These components are adapted from different frameworks, creating platforms for multidisciplinary research (Nuijten, 2011). The different components are outlined below, highlighting the limitations they address and the elements incorporated from existing frameworks.

Drawing on Scott’s (2006) Vulnerability framework, *Environment* incorporates climate hazards (sudden shocks, long-term stresses, seasonality shifts), and landscape characteristics such as landcover and connectivity (i.e., proximity to road networks). Incorporating these elements under the umbrella term *Environment* also reflects how climate hazards can influence landscape characteristics; for example, flooding along major roads may exasperate physical remoteness (Amankwaa & Gough, 2023), or coastal erosion may remove cultivated landcover (Baffour, 2024). Landscape characteristics may also influence climate hazards; for example, non-permeable built-up landcover may induce greater flooding during heavy rains (Abass et al., 2020). Landscape characteristics also contain information on different ES types (Kullo et al., 2021), therefore, the framework links 'landscape' to 'natural capital/perceptions' to reflect how *Environment* can be interpreted as a wellbeing determinant, through its contributions to people (Hill et al., 2021), and/or constituent depending on the research context (Schleicher et al., 2018).

The framework’s underlying foundation is the IPCC risk framework, with hazards influencing OWB and SWB differently depending on exposure and vulnerability (Cardona et al., 2012). Including *Exposure* acknowledges environmental conditions to differ across space. For example, deltaic communities are more exposed to flooding due to low-lying land (Van Soesbergen et al., 2017). *Exposure* is a component, yet not a determinant, of risk (IPCC, 2024). Depending on a household’s sensitivity and adaptive capacity, they may be exposed yet not vulnerable to hazards.

*Wellbeing* draws on the SLF capitals to incorporate different resources which control livelihood strategies, and are depended upon to “durably sustain people’s basic needs” (Gaillard et al., 2009; p.120). Five objective capitals (financial, human, social, natural, physical) highlight the different, yet interconnected, elements of OWB. The capitals are mirrored by subjective perceptions to reflect the potential interdependence between OWB and SWB (Atkinson, 2013). For example, cultural ESs (natural perception) are produced through subjective interpretations of objective natural capital (i.e., aesthetic landscapes) (Buchel & Frantzeskaki, 2015). Previous research demonstrates SWB to be sufficiently sensitive to capture wellbeing differences across diverse landscapes and agricultural systems, “supporting the…inclusion of such measures in sustainability frameworks” (Brown et al., 2021; p.1). Exemplar measures for each capital and perception are presented in Figure (1).

Depicting *OWB* and *SWB* as distinct yet interconnected aligns with WeD, broadens potential research avenues, and addresses limitations of SLF which focuses on objective, quantitative checklists and does not explicitly capture qualitative, subjective meanings attached to resources or assets. Furthermore, from a policy perspective, including paralleled OWB and SWB prevents one from being a substitute for the other, reducing the risk of “happy but poor” communities being excluded from material support (Davis, 2014). Including broad capitals and perceptions also provides an overarching structure which can be flexibly interpreted according to community experiences, the research aims and available data (Fisher et al., 2013).

By positioning the capitals and perceptions under the umbrella term *Wellbeing*, the framework illustrates how the different elements can positively and/or negatively influence each other, either directly (i.e., education [human capital] improving access to higher-paid employment [financial capital]) or indirectly by shaping adaptive capacity and/or sensitivity. Including positive and negative relationships provides equitable opportunities for different actors’ experiences to be captured (Luque-Lora, 2024). For example, access to community groups (social capital) may increase individuals’ financial capital due to collective saving schemes (Di Falco & Bulte, 2009), while climate-induced reductions in financial capital may conversely increase community cohesion (social perception) (Lent, 2004). The framework’s flexibility to examine non-tangible dimensions, such as social cohesion, can advance understanding of how climate hazards drive demographic change and impact the capacity for collective adaptation (Husile, 2025). Furthermore, unlike frameworks assuming uniform influences on wellbeing, such as TEEB (2010) which depicts human wellbeing as a single component, the ECW framework allows OWB and SWB to respond and interact differently, representing a key contribution of the framework.

By recognising relationships between capitals and perceptions, and allowing indicators to be classified differently according to local context, the framework addresses Gough and McGregor’s (2007) critique of frameworks that confine assets to single dimensions (i.e., SLF). Nevertheless, from a policy perspective a balance is required between flexible frameworks which capture local complexities, and structured models which improve interpretability, facilitate analytical comparability, and ensure key elements are not overlooked (Loveridge et al., 2020).

*Wellbeing* is depicted as dynamic within time and space (see *Relational context*). Unlike WeD, which views wellbeing exclusively as a process, the ECW framework interprets it as an outcome and process. Outcomes are not endpoints, they can be reshaped or contribute towards further goals. However, from a policy perspective, wellbeing must be a quantifiable outcome that can be monitored (Sachs, 2019). Furthermore, the framework views *Wellbeing* at different scales, for example physical capitals may contain household assets or community-wide infrastructure, while subjective perceptions offer opportunities for qualitative research into individual-level perspectives; challenging the traditional dominance of quantitative methods in sustainability studies (Althor & Witt, 2020). In particular, by incorporating social capitals and perceptions, and acknowledging the Relational context, the framework supports research in locations where wellbeing is experienced individually and collectively. The framework’s cross-scale flexibility represents a key contribution, given that much existing work focuses on individual outcomes despite growing recognition that climate hazards can have cumulative impacts for households and communities (Piringer et al., 2024). For example, ill-health of one household member may increase caregiving burdens for others, thereby reducing their financial productivity (Cuartas et al., 2025).

Many studies adjoin *Adaptive capacity* and *Sensitivity* within ‘vulnerability’ (Cardona et al., 2012; Scott, 2006); however, the ECW framework differentiates them to illustrate the two channels through which environmental conditions impact Wellbeing and Response. Weis et al. (2016) define *Sensitivity* as the “characteristics of a community that influence its likelihood to experience harm under a given stressor” (p.620); for example, low-quality housing (physical capital) may be damaged more severely by intense precipitation (Atiglo & Codjoe, 2017). This definition suggests objective assets and resources control *Sensitivity*. However, the ECW framework shows subjective perceptions to also influence *Sensitivity*; for example, communities with stronger affiliation to nature (natural perception) may experience disproportionately larger SWB reductions following degradation (Albrecht et al., 2007).

*Adaptive capacity* refers to the ability to adjust or take advantage of changing conditions (IPCC, 2022); incorporating “asset-based components [objective capitals]…and less-tangible aspects such as flexibility [and] innovation [subjective perceptions]” (Weis et al., 2016; p.620). For example, education (human capital) increases access to climatic information for long-term risk planning (Muttarak & Lutz, 2014), whereas low subjective financial stress increases the willingness to invest in adaptation during environmental pressure (Cooper et al., 2008; Rashid et al., 2020). These examples counter Nyamwanza‘s (2012; p.3) assertion that “adaptive capacity is largely a function of [objective] resources and assets”.

Objective capitals and subjective perceptions are included as wellbeing ends, and as “part[s] of a process towards other desirable goals” (Atkinson, 2013; p.139) by controlling *Adaptive capacity* and *Sensitivity* (Little et al., 2001). For example, collective strategies may produce win-win outcomes by increasing SWB, social cohesion and empowerment, while also addressing objective environmental challenges (Lawrance et al., 2022). How capitals and perceptions influence vulnerability may depend on the research context. For example, in subsistence communities, education (human capital) may be more influential in increasing *Adaptive capacity* by improving agricultural practices, rather than reducing *Sensitivity* through the provision of non-traditional livelihoods (Brown et al., 2021). This approach contrasts SLF which only incorporates capitals as means to wellbeing (Fisher et al., 2014), and does not explicitly include *Adaptive capacity* or *Sensitivity*. It also differs from Scott’s (2006) Vulnerability framework which suggests capitals only mediate *Sensitivity*. Therefore, the ECW framework adds value to environment–wellbeing research and interventions by situating wellbeing outcomes within broader targets for reducing environmental vulnerability.

The framework also illustrates how Wellbeing, and therefore *Adaptive capacity* and *Sensitivity*, can influence Exposure and Environment. Both elements also link to what Response is taken to cope or adapt to environmental challenges. For example, households without safe latrines (physical capital) may be more sensitive to flood-induced disease, and therefore respond by constructing flood defences. Moreover, by acknowledging OWB and SWB to produce different levels of *Adaptive capacity* and *Sensitivity*, the framework recognises social differentiation in resources, rights and capabilities; as noted in EEE (Fisher et al., 2013).

As outlined in SLF, objective capitals govern *Livelihood* practices (Scoones, 1998). For example, education (human) and land rights (natural/physical) may influence rural workers’ land management strategies (Muttarak & Lutz, 2014). The framework also illustrates how subjective perceptions influence *Livelihood* choices; for example, farmers fulfilling intergenerational identities (Brown et al., 2021; Miñarro et al., 2022). Furthermore, by connecting *Livelihood*, OWB and SWB, the framework acknowledges livelihood type to shape how Wellbeing is conceptualised and what capitals or perceptions are desired (Appadurai, 2004; Dzokoto et al., 2019).

*Livelihoods* also control Sensitivity. For example, while entire communities may be exposed to drought, it may only constitute a risk to farmers due to the interconnectivity between ESs, food security and health (Kuenzer & Renaud, 2012). Therefore, incorporating *Livelihoods* supports research on how similar hazards may impact households’ OWB, SWB, and Response differently. By recognising wellbeing capitals and perceptions to govern *Livelihood* practices, the ECW framework is novel in encompassing elements from livelihood and wellbeing approaches (Nunan, 2015). This approach permits the exploration of broad, quantifiable trends and in-depth, individual-level values.

As outlined in DPSWR, SLF and Vulnerability frameworks, the ECW framework explicitly incorporates *Response* to acknowledge actors’ agency to respond to environmental and wellbeing changes. This counters vicious circle theories which assume environment-wellbeing relationships are inevitably negative (Reardon & Vosti, 1995). Individuals’ wellbeing capitals and perceptions influence *Response*. For example, long-term migration requires extensive financial, social and human resources (i.e., access to job opportunity information) (Flahaux & De Haas, 2016). *Response* incorporates short-term coping (Chambers & Conway, 1992), referring to reactionary practices within existing structures, and long-term adaptations (livelihood or environmental) which address broader contextual challenges (Adger et al., 2003). Adaptation ranges from soft educational or ecological strategies with minimal physical change, to hard technological measures which change infrastructure or markets (Dovie, 2017); for example, coastal defences or livelihood diversification (Kuenzer & Renaud, 2012). *Response* can also feedback to influence Wellbeing and Livelihood; for example, adapting to drought by introducing new farming technologies and practices (Funk et al., 2020).

Adopted from MEA, TEEB, SLF and DPSWR, *Global drivers* acknowledge the broader economic, social, political, demographic and historical contexts which influence wellbeing. For example, global market demands influence which crops are grown and the price received (MEA, 2005). Incorporating this component helps prevent environmental and wellbeing challenges from being interpreted as internally caused and experienced. Acknowledging macroscale *Global drivers* therefore supports cross-scale research by situating local perspectives within overarching structures (Newton, 2007).

Environment-wellbeing relationships exist within the *Relational context*, meaning they are not formed in a vacuum (Tosam & Mbih, 2015), but are situated within specific spatiotemporal settings (White, 2010) and shaped by historical power relations (Ayanoore & Hickey, 2022; Smith, 2021). This perspective aligns with the IPCC risk framework which views “vulnerability and exposure [as] an outcome of skewed development processes”, including uneven investment patterns and natural resource mismanagement (Cardona et al., 2012; p.70). It also incorporates thinking from EEE, SLF and TEEB, which highlight how authoritative institutions and natural resource governance structures shape the capacity to translate environmental resources into wellbeing outcomes. The *Relational* perspective upholds principles of recognitional justice (Calderón-Argelich et al. 2021) and cultural awareness by acknowledging differences in how wellbeing is conceptualised and experienced across sociodemographic groups, and ensuring external assumptions are not uncritically applied across various settings (Fadiji et al., 2021). Wellbeing is not passively experienced, but actively constructed in time and space.

Framing OWB and SWB within the *Relational* supports the ‘comprehensive’ approach, recognising the “psychological linkages between the person and [their] membership groups” (Lent, 2004; p.489). Connecting the individual and collective is pivotal within LMIC research, where wellbeing can often be achieved through social interactions and reciprocal relationships (Schaafsma & Gross-Camp, 2021). For example, agricultural tools (physical capital) may support food security (human capital), yet also boost SWB in contexts where fulfilling obligations to provide for the family are valued (Markussen et al., 2018). Additionally, by acknowledging the temporal context, the framework supports research on how climate and landscape changes may reshape how wellbeing is experienced over time.

**Fig. 2:**
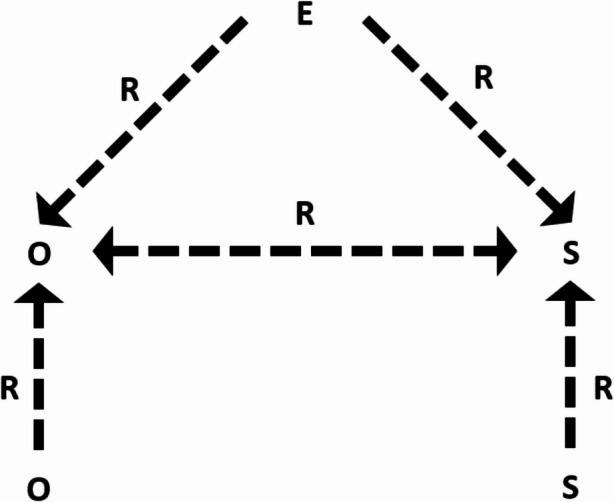
Visualisation of how the relational (R) acts as a mediating process within time and space, influencing the relationships between OWB (O), SWB (S) and environment (E)

The framework interprets the *Relational* as a mediating process that shapes how different OWB and SWB elements are valued, associated, and influenced by environmental conditions (Fig. 2). This approach contrasts WeD which incorporates *Relational* as a wellbeing component alongside subjective and material wellbeing (White, 2010), and TEEB (2010) which places socio-cultural wellbeing as a static outcome. This interpretation of the *Relational context* bridges the gap between OWB and SWB; supported by Atkinson (2013) who suggests that relationships between wealth, health and happiness are influenced by social norms and what is achievable within society at a given time. Figure (2) illustrates this interconnected relationship; for example, the extent to which climate hazards (E) impact SWB (S) may be influenced by the temporal context (R) (i.e., if similar events occurred before). Spatial differences in public and private investment (R) may also affect the value attached to financial capital (Guillen-Royo & Velazco, 2012); therefore, climate hazards which reduce economic activity (O) may have larger SWB impacts (S) in certain contexts. 

Figure (1) provides an overview of key wellbeing elements; however, which capitals and perceptions are included within research depends on the *Relational context*. Furthermore, to minimise visual complexity, Figure (1) depicts the *Relational context* as a broad, all-encompassing concept across the environment-wellbeing system. Yet, in practice, the *Relational context *sits within each dynamic arrow within the framework, mediating how different wellbeing elements are valued and related.

The *Relational* view ensures research supports policy in incorporating “people’s multiple and context-specific ways of valuing nature [and wellbeing]” (Arias-Arévalo et al., 2018; p.1), and discourages ineffective blanket approaches (Walsham, 2020). For instance, policies assuming unanimous benefits from cropland expansion may reduce SWB if communities’ identities are attached to pre-existing landscapes (Gross-Camp et al., 2019). The importance of acknowledging *Relational context* when implementing interventions, assessing community behaviours (Gross-Camp, 2017), and evaluating local policy buy-in is illustrated by Coulthard’s (2011) study of South Indian fishing communities. Initial results, using the macroscale MEA framework, assumed increased financial wellbeing improved adaptive capacity through livelihood diversification, yet follow-up work using the WeD approach unearthed how affluent households were not diversifying due to fears that moving from specialisation to diversification would reduce social prestige. This example highlights the differences between livelihood and wellbeing approaches (Nunan, 2015), and emphasises the importance of exploring environment-wellbeing relationships through a *Relational* lens.

While the *Relational context* is primarily interpreted through qualitative analysis, future research could employ innovative mixed-method strategies. Sequential approaches (Creswell & Creswell, 2017) can allow communities’ diverse characteristics, livelihoods, and lived experiences to inform contextually relevant wellbeing data collection. For example, Q-methodology, which combines quantitative analysis of participants’ values with in-depth qualitative data (Kabir et al., 2024), could support research using the ECW framework by identifying how groups with varying wellbeing priorities, clustered using factor analysis, experience climatic risks differently. The *Relational context* can also be operationalised within quantitative environment-wellbeing models by controlling for individual and contextual factors, and incorporating interaction terms with environmental variables to explore how different characteristics moderate environmental impacts on wellbeing. Where appropriate, spatial or multilevel models can further capture context-specific variation in wellbeing across communities beyond that explained by measured variables (Feaster et al., 2011).

Overall, the ECW framework is innovative in incorporating elements from wellbeing and livelihood frameworks (Nunan, 2015), supporting research on interactions between OWB and SWB, and acknowledging how environmental conditions and the Relational context impact these relationships differently over spatial and temporal scales.

## Case study (Volta Delta, Ghana)

### Study summary

The ECW framework supports comprehensive wellbeing research in environmentally vulnerable contexts. To illustrate the framework, a case study from Volta Delta, Ghana is presented. The study explores relationships between environmental conditions, OWB and SWB across different landscapes.

**Fig. 3:**
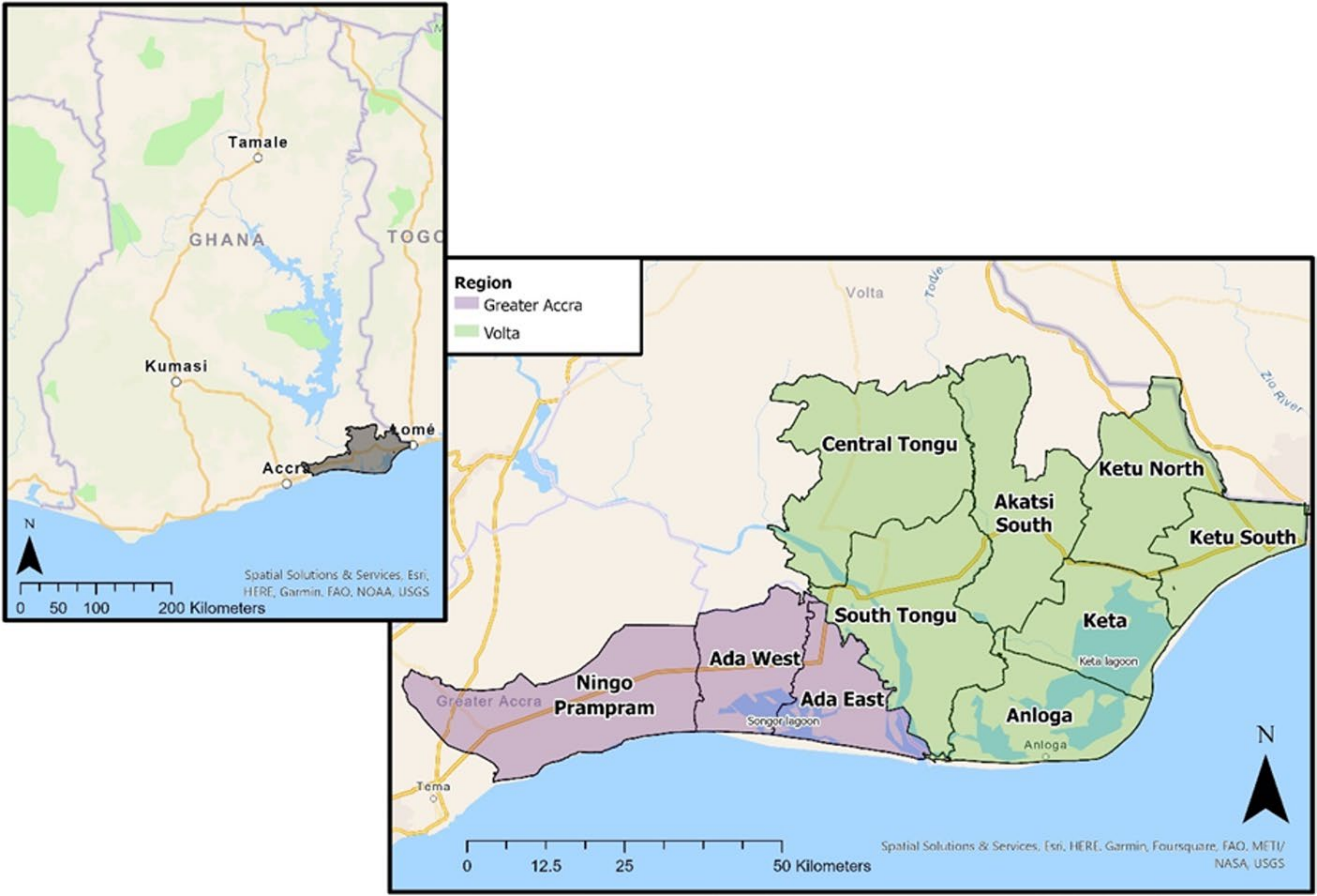
A map of Volta Delta, with regional and district boundaries

Volta Delta is located across Volta and Greater Accra regions (Fig. 3). Landcover predominantly consists of cropland, grassland, and wetland. Approximately one-third of individuals work within agriculture, higher than the proportion of GDP generated by agricultural activities (22%) (Addo et al. 2018b; Cazcarro et al. 2018). The disparity is driven by subsistence farming, low productivity and limited technology access (Arto et al., 2020). The prominence of subsistence agriculture accentuates the interconnectivity between livelihoods, wellbeing and environment (Duku et al. 2022b). Volta Delta is exposed to multiple climate hazards, including droughts, uncertain seasonality, coastal flooding, erosion, and land/aquifer salinization (Addo, 2015; Codjoe et al., 2020).

The mixed-method study included binary logistic regression models (Appendix 5), using Deltas, Vulnerability and Climate Change: Migration & Adaptation (DECCMA) survey data, exploring environmental relationships with OWB, SWB, and aligning/opposing outcomes (Table 1). The survey was undertaken face-to-face with household heads (April-June 2016). A two-stage clustered sampling strategy attempted to survey 1,500 households. Households were stratified into five strata based on environmental risk. Fifty enumeration areas were randomly selected proportional-to-size from the strata. Thirty residential dwellings from each enumeration area were randomly selected. A 91% response rate was achieved (1,360 households). See Atiglo et al. (2022) for further information on the survey strategy.

**Table 1:** The eight binary logistic regression models developed from DECCMA survey data and remote sensing variables. See Appendix (5) for the full models; referenced using the model number

Outcome type	Model number	Dependent variable
Objective	1	Expenditure poverty
	2	Basic needs deprivation
Subjective	3	Financial stress
	4	Unhappiness
Aligning	5	Non-poor & Happy
	6	Poor & Unhappy
Opposing	7	Non-poor & Unhappy
	8	Poor & Happy

See Appendix (6) for the methodologies used to calculate the dependent variables

Participatory rural appraisal methods across eight communities were also conducted (Appendix 7), including focus groups (FGs), semi-structured interviews, and weighting exercises capturing communities’ wellbeing priorities (Cannings et al., 2025a). Communities were selected based on key themes from quantitative modelling, including areas with distinct wellbeing outcomes, landscapes and climatic characteristics. Interviews with eight District Planning Officers (DPOs) also captured their perspectives on communities’ environmental experiences and the impacts on different wellbeing elements.

### Case study results

The following section summarises key findings from the case study to support the framework’s components^4^. Across the eight core framework components, thirteen key relationships are discussed (numbered in Fig. 1). The ECW framework is not intended to guide research toward capturing all elements of the socioecological system; rather, it serves as an analytical tool to simplify complex phenomena and ensure key elements are not overlooked. Although the components are interconnected, the framework can support analysis of specific relationships aligned with different research scopes while still ensuring contextual factors and feedback effects are acknowledged (Nunan, 2015). See Appendix (8) for two illustrative examples of the interconnected components in the full framework.

Firstly, quantitative model results illustrated climate hazards and landscape characteristics to significantly associate with both OWB outcomes, supporting *Environment* as a wellbeing determinant (Fig. 1, **item #1**). For example, residing near drought-exposed grasslands increased the odds of expenditure poverty (Appendix 5), supported by qualitative respondents discussing their financial losses, “*last year I cultivated two acres of cassava and it rained generously so I had…15–20 sacks*,* with this I invest 2*,*000 cedis. But I cultivated the same acres this year and got 3 bags…the money is totally lost*” (Male interview). 

Quantitative models also illustrated *Environment* as a SWB determinant (**item #2**). Changing rainfall patterns increased odds of financial stress, while impacts from salinisation increased unhappiness (Appendix 5). These themes were supported by respondents noting how reduced rainfall brings emotional challenges, “*drought leads to poor yield which brings heartache*” (Male FG), and coastal hazards disturb peace, “*when the sea becomes violent…*p*eople are displaced from their homes…you become disorganized…when you [move to] somebody’s house you will not have peace”* (Female FG). The second quotation also highlights the underresearched interconnectivity between climate risk, wellbeing and demographic dynamics (i.e., household agglomeration) (Piringer et al., 2024). 

Respondents also noted how hazards exacerbate inequalities and relative comparisons to others, key SWB controls (Ravallion, 2014). For example, persistent flooding in Ada West (Fig. 3) resulted in investment being channelled to the nearby district capital (Sege), creating visible inequalities; “*the big houses in Sege are owned by people in Anyamam. They use Anyamam as a business point and do their fishing…but in terms of assets they do it [in Sege]…they know anytime the floods can take over”* (DPO interview).

The ECW framework also interprets *Environment* as a wellbeing constituent (Schleicher et al., 2018), illustrated in the framework (Fig. 1) by the connection between 'landscape' and 'natural capital/perceptions' (**item #3**). This interpretation was captured through qualitative methods, with respondents framing natural landscapes as fundamental components of their ‘good life’; “*this is the land our forefathers reserved for us…we are excited about where we have*” (Female FG), “*good life and happiness exists through the fresh air we get here*” (Male FG).

Certain communities and livelihoods were located in areas with greater *Exposure* (**item #4**); for example, one respondent noted “*if you are unfortunate to have your work located at the shores of the sea*,* you’ll always be in fear”* (Male FG). Similarly, inland crop farmers recorded greater *Exposure* to drought and water insecurity; “*unlike our counterparts closer to the sea*,* they easily access water unlike us who depend on God-given rain*” (Male FG).

The ECW framework appends *Livelihoods* to OWB and SWB to illustrate how wellbeing is conceptualised differently across groups (**item #5**). Weighting exercise results illustrated significantly different wellbeing priorities across livelihood groups (Cannings et al., 2025a). For example, farming communities weighted “employment” and “cooperative membership” higher than fishing and peri-urban groups. The influence of *Livelihood* and local context is further illustrated by respondents’ contrasting responses to “who lives the best life?”. In Ningo Prampram, near the industrial hub Tema, a respondent stated those doing the best “*are people who have a lot of money*” (Male interview), whereas in rural Akatsi South a respondent defined success through crop quality (Female interview).

*Livelihoods* also influence *Sensitivity* (**item #6**); with quantitative models reporting higher odds of expenditure poverty, basic needs deprivation and financial stress amongst crop farmer and fishing/trade groups, compared to business owners/salaried employees (Appendix 5). These results suggest primary livelihoods, involving the extraction of raw materials, are more sensitive to environmental challenges. This is reflected by urban respondents near Tema and Accra transitioning away from farming to minimise financial uncertainty; “*our youths are moving to the industrial sector and abandoning the agricultural sector*” (DPO interview). In contrast, those with fewer opportunities experienced financial challenges; “*farming is our only source of income…the rainfall patterns are not helping our yields*,* which reduces the income we get from selling*” (Male FG).

OWB and SWB are separate, yet interconnected, components (Cannings et al., 2024) which positively and/or negatively influence one another (“+/-“). Firstly, exploring reinforcing mechanisms (**item #7**), quantitative models showed aligning “non-poor/happy” outcomes to associate with stable climates, including consistent rainfall levels and limited erosion (Appendix 5). Greater certainty in current and future conditions can increase the confidence to invest in objective living standards (Cooper et al., 2008) and reduce feelings of powerlessness (Stokols et al., 2009). Respondents from Akatsi South also illustrated positive relationships between land access (natural/physical capital) and autonomy (human perception), a key element of SWB (Deci & Ryan, 2012); “*over here the land is free*,* we have built our houses*,* they are for us and not anyone else…We are content because no one is chasing us for rent*” (Female FG). Financial capital also positively associated with social capital, with many noting how monetary resources are central to forging and strengthening communal relationships in rural Ghana (Dzokoto et al., 2019; Tsai & Dzorgbo, 2012).

Mixed-method results also supported opposing relationships between capitals and perceptions (**item #8**). For example, higher odds of “poor/happy” outcomes associated with agricultural landscapes (Appendix 5). Higher SWB potentially emerged due to remoteness from urban areas limiting relative comparisons to higher living standards (Ravallion, 2014), “*they only compare themselves to others in the community*,* they cannot compare to others because they are all subsistence farming”* (DPO interview), and the lower value placed on material components (Ward & King, 2016);*“even though I am not rich I am happy and I do not want to overburden myself thinking about my lack of money”* (Male FG).

In contrast, “non-poor/unhappy” outcomes associated with peri-urban communities exposed to flooding (Appendix 5). Despite higher OWB, driven by less-vulnerable livelihoods and greater public investment, SWB was hypothesised to be lower due to higher inequality (Guillen-Royo & Velazco, 2012) and fears of coastal hazards (Sekulova & van den Bergh, 2016); “*they worked hard and accumulated all those things and the rain just…washes it all away…If you are not careful the person will overthink things and die*” (Female FG). However, negative associations also produced positive outcomes, with respondents reporting increased community togetherness (social perception) when flooding destroyed dwellings (physical capital).

The framework depicts Wellbeing as an outcome and as a means to further Wellbeing by controlling *Sensitivity* (**item #9**). For example, quantitative results illustrated an interaction between households’ water infrastructure (physical capital) and “distance to water source” (landscape), suggesting piped water access minimised *Sensitivity* to expenditure poverty amongst remote households (Appendix 5). The interaction may reflect lower time poverty among household members with domestic responsibilities; “*if I have to do chores; sweeping*,* fetching water*,* washing…which take up a lot of time it will not be possible [to do business]*” (Female interview).

The framework also illustrates SWB to influence *Sensitivity*, supporting Addai et al. (2014) who claim happiness creates a resiliency culture. For example, lower odds of unhappiness amongst agricultural households with high place attachment (Appendix 5) suggests rural communities’ affiliation with their landscape and community may act as compensatory factors, reducing the *Sensitivity* of SWB to environmental vulnerability and material deprivation (Milbourne, 2014). However, qualitative discussions suggested this relationship is multidirectional, with place attachment (social/natural perception) potentially intensifying subjective harm; “*The attachment…this is where I’m born…our fathers suffered in building a house only to have it destroyed by the sea…this brings depression*” (Male FG).

OWB and SWB are also interpreted to influence *Adaptive capacity* (**item #10**), supported by multiple qualitative examples. Respondents highlighted the importance of education (human capital) and credit (financial capital) in facilitating adaptive farming (i.e., irrigation) and improving construction practices, the latter mitigating unplanned financial costs and the negative SWB impacts associated with being unable to maintain one’s home (van der Geest, 1998); “*because of education we now use slate roofing. The sea breeze cannot destroy it…We are no longer embarrassed by not being knowledgeable*” (Female FG). Family and friend networks (social capital) also increased *Adaptive capacity* by providing soft loans or farming advice; “*a friend in the next town…I admire their work…[he told me]…buy this cow dung*,* spread it on the ground and plant. They encourage me and gave me guidance*” (Male interview). SWB elements also influenced *Adaptive capacity*, with residents recording how place attachment (natural/social perception) stimulated action to protect their land from coastal erosion; “*some of us held strong…we are still here protecting the little left for the future*” (Male FG).

Quantitative results highlighted the importance of *Response* to agricultural communities **(item #11**), with rural households undertaking adaptive practice(s) exhibiting lower odds of expenditure poverty (Appendix 5), reinforcing evidence that adaptation often contributes more towards poverty alleviation than raising average incomes in Sub-Saharan Africa (Heger et al., 2018). The feedback effect from *Response* to Wellbeing was also supported by farmers highlighting how irrigation reduced Sensitivity to drought, and improved financial OWB by providing a market advantage over rain-fed farmers; “*the farmers are waiting for rains*,* so there is not as much of a supply*,* so when they hear we have okra here they come rushing*” (Male interview).

However, similar to the mixed associations between capitals and perceptions, inconsistent adaptation outcomes were also illustrated. For example, households in Greater Accra with past migrant experience associated with lower odds of basic needs deprivation (Appendix 5), potentially reflecting greater access to financial, knowledge and social remittances from Tema and Accra (Awumbila et al., 2017; Codjoe et al., 2017). Yet, across Volta Delta past migration associated with both low financial OWB and SWB (Appendix 5), suggesting potential maladaptation. Lower SWB may have resulted from demographic shifts lowering social cohesion (Husile, 2025), or from migrants’ exposure to higher living standards increasing the visibility of unobtainable reference points (Knight & Gunatilaka, 2010); “t*hose [in Tema/Accra] have more money than us…they have their bodies looking nice*,* but us over here we are not looking nice”* (Female FG). Similarly, higher odds of expenditure poverty among households with current migrants (Appendix 5) may reflect how emigration increases pressures on origin communities; “*migration is disadvantaging us…instead of producing to feed Accra*,* we now go to Accra to buy foodstuffs”* (DPO interview). The association with low OWB and SWB emphasises how effective adaptation and migration policy must account for positive and negative feedbacks, and the material, “psychological, symbolic and emotional aspects” (Adger, 2010; p.282).

Quantifying *Global drivers* (**item #12**) remains challenging, justifying the use of participatory qualitative methods in environment–wellbeing research. Nonetheless, certain results could be interpreted through a global lens. For example, lower odds of basic needs deprivation in Greater Accra compared to the Volta region (Appendix 5) may reflect historically uneven investment and disparities in human capital, infrastructure, and livelihoods (Kambala, 2022). Similarly, the prominence of vulnerable agricultural livelihoods in Volta Delta can be linked to *Global drivers*, with structural readjustment programmes generating development pathways centred on the unsustainable extraction of exportable materials (Atta-Quayson, 2023). 

Similar to Global drivers, *Relational context* was also interpreted through qualitative findings to help refine quantitative results (**item #13**). The case study highlighted a distinction between collective agricultural communities and individualistic peri-urban towns (Cannings et al., 2024). Long-term differences in private and public investment may have resulted in personal success and financial wealth being prioritised in built-up areas, compared to the emphasis on community relationships within rural areas accustomed to slower socioeconomic development (Pokimica et al., 2012); “f*or urban dwellers their focus is on making money…meanwhile in rural areas*,* money is not their goal*,* their goal is having the social network*,* the communal feeling*” (DPO interview). Such collectivism in rural areas may foster psychological resilience to hazards, helping maintain higher SWB; “*even if there is drought*,* people are still happy*,* the relationships are still there despite the hard times”* (Male interview). In contrast, higher odds of financial stress in built-up areas exposed to storms (Appendix 5), could reflect how urban individuals perceive themselves as having more-to-lose, resulting in greater climate-induced SWB reductions. These examples support Figure (2) by showing the *Relational* to mediate the value attached to financial capital (OWB), and the extent to which environmental impacts on OWB influence SWB.

The *Relational context* also captures the influence of power relations and natural resource governance on the environment-wellbeing system. For example, the government’s sale of the 41,000-acre Songor lagoon in Ada West to private industry removed local communities’ traditional salt mining livelihoods^5^. Communities’ financial OWB and SWB were lowered as no alternative occupations were provided, new forms of individual ownership disrupted collective social norms, and violent clashes between resistant communities and private police reduced perceived safety (Agbove, 2022; Yeboah et al., 2013); “*you don’t have the right to take salt out of the lagoon*,* this has brought war between the town people and the individual…affecting our peace in the community*” (Male FG). Despite proximity to profitable salt resources, relational structures limited actors’ capabilities, and the associated financial and cultural benefits. These examples underscore the importance of accounting for *Global drivers* and *Relational context* in local research to challenge external assumptions and avoid interpreting environmental and wellbeing challenges as internal processes.

### Challenges

The case study also challenged certain elements of the ECW framework. Firstly, the terminology *Wellbeing* provides a positive and holistic outlook, removing assumptions that sustainable development is solely for deprived groups. However, this could cause negative outcomes to be overlooked. The ECW framework uses *Wellbeing* for positive and negative impacts, whereas others differentiate well/ill-being (White, 2010).

In contrast, drawing on the IPCC risk model arguably suggests climate hazards are universally harmful (Reisinger et al., 2020). However, multiple respondents noted positive or neutral impacts. For example, accepting drought as “*God’s will*” dampened SWB impacts; “*drought has become part of their lives*,* whether it rains or not*,* they stay in the same position*” (Male FG). Others also noted the sense of achievement from overcoming or surviving climatic challenges; *“Good life means at first you faced hardship*,* but after a while there have been some changes and you are no longer facing hardships”* (Female FG). Nevertheless, by framing *Adaptive Capacity* and *Sensitivity* within *Wellbeing*, rather than explicitly defining them as elements of risk, the ECW framework allows negative, neutral and positive impacts to be explored.

Certain findings also disputed assumptions that *Wellbeing* consistently improves *Adaptive capacity* and/or *Sensitivity.* For example, high place attachment (natural/social perception) did not significantly reduce the odds of expenditure poverty when impacted by drought (Appendix 5). Respondents also noted how education (human capital) may conversely raise expectations, which if unmet due to the impacts of climate risk or the systemic disconnect between education and job markets, can lower SWB. Therefore, the framework should be applied flexibly to capture both positive and negative influences.

Participants also frequently proclaimed “money is blood”, referring to how money maintains the fabric of Ghanaian culture, health and relationships (Hasty, 2005). Therefore, depicting decomposable wellbeing elements may not align with local understandings of financial capital as being indistinguishable from other elements of a ‘good life’. However, encompassing different capitals and perceptions under the umbrella term *Wellbeing* illustrates their capacity to coexist. Moreover, a framework must offer practical support to research and policy, with defined categories improving results’ simplicity and interpretability (Nunan, 2015).

Conclusively, the ECW framework contains multiple complex interactions compared to other frameworks (Appendix 3). While the complexity and inclusion of local contextual effects can present challenges for data collection and generalisability, they prevents the artificial oversimplification of the environment-wellbeing system, which could otherwise lead to one-size-fits-all interventions which entrench inequalities and fail to address groups’ specific needs (Ostrom, 2009). Furthermore, research using the ECW framework need not examine the entire complex system; rather, it can be used to identify key variables and relationships for further analysis (Nunan, 2015).

## Conclusion

The ECW framework supports research on relationships between environmental conditions and comprehensive wellbeing. While existing frameworks incorporate similar ideas, this framework explicitly focuses on climate risks and landscapes, while acknowledging the capacity for multiscale influences and different conceptualisations across relational contexts. Incorporating OWB and SWB encourages mixed-method research to capture local voices and context-specific priorities, alongside universal sustainable development objectives.

The framework can be applied flexibly to capture positive, neutral or negative impacts from climate risks and landscape changes. For example, the case study illustrated how certain wellbeing elements do not universally lower vulnerability. Therefore, the depicted interrelationships should be examined within the relational context to critically evaluate commonly held assumptions about environment–wellbeing relationships.

The framework is primarily designed to explore environment-wellbeing associations within rural LMICs; however, it can be used to compare different locations, or identify local relationships that differ from documented associations. Depending on the relational context and global drivers, research may prioritise different capitals and perceptions that are relevant to target communities. Accordingly, the framework can guide participatory data collection, such as weighting or ranking exercises (Cannings et al., 2025a), to develop contextually relevant wellbeing measures. This application could address a potential limitation of the ECW framework whereby presenting OWB and SWB in parallel could imply equal importance, despite SWB often being viewed as a ‘bonus’ compared to pressing material needs in low-income communities (Chumo et al., 2023). The breadth of capitals and perceptions also allows researchers to flexibly integrate the elements available within different datasets. 

The ECW framework emphasises the importance of viewing environment-wellbeing relationships within their spatiotemporal context, recognising how actions and resources at various scales and time periods influence local, current-day outcomes, and how OWB and SWB interact. For example, the dichotomy between high OWB-low SWB individualistic peri-urban communities, and low OWB-high SWB collective rural neighbourhoods (Cannings et al. 2024). Furthermore, future environmental conditions will inevitably change, creating new or reshaping existing relationships. Therefore, the framework could inform longitudinal studies to explore how conceptualisations of wellbeing and environmental relationships change with evolving global drivers, climate hazards, and demographic dynamics.

The ECW framework situates ‘wellbeing’ within the broader concepts of risk and vulnerability by interpreting it both as an outcome and as a process which influences adaptive capacity and sensitivity. This approach embeds human wellbeing, and its multiple interpretations and local nuances, within policies addressing environmental challenges, reducing the risk of negative trade-offs that arise when environmental outcomes are targeted in isolation (Angnuureng et al., 2023). By allowing environmental conditions to influence capitals and perceptions differently, the ECW framework enables the analysis of interconnected trade-offs within socioecological systems, a research avenue not afforded by frameworks with unidimensional or narrowly defined OWB factors.

The incorporation of SWB, which distinguishes the ECW framework from most prominent environment-wellbeing frameworks (Schleicher et al., 2018), also has important policy implications by creating channels for public voices and facilitating actor-orientated approaches (Roy et al., 2011). As illustrated in existing studies (Coulthard, 2011; Gross-Camp, 2017) and the Volta Delta case study, individuals’ emotions and perceptions govern behaviour. Therefore, evaluating how environmental challenges and policy decisions influence SWB can improve decision-makers’ understanding of communities’ responses, and support the development of sustainable interventions to enhance environmental and human wellbeing.

## Supplementary Information

Below is the link to the electronic supplementary material.

Supplementary Material

## Data Availability

The interview and focus group transcripts are available via request here (10.5258/SOTON/D3432). The DECCMA survey data used for the statistical analysis is openly available here (https://data.mendeley.com/datasets/223z53kwnm/1).

## Footnotes

1 Sustainable Development Goal 3: “Ensure healthy lives and promote wellbeing for all”.

2 Sustainable Development Goal 2: “End hunger, achieve food security and improved nutrition and promote sustainable agriculture”.

3 While Nunan’s (2015) work addresses poverty and the environment, the concept of 'poverty' is conceptualised broadly and should not be narrowly interpreted as objective or financial wellbeing.

4 For more information on the related studies, please see (Cannings et al., 2024, 2025a, 2025b).

5 Following the 2006 Minerals and Mining Act, salt was classified as a state-owned “mineral” (Parliament of the Republic of Ghana, 2006).
