# Supplementary material for: An environment and comprehensive wellbeing (ECW) conceptual framework: exploring environmental relationships with objective and subjective wellbeing
Source: Popul Environ. 2026 Mar 28;48(2):6. doi: 10.1007/s11111-026-00518-w (PMC13032941; doi:10.1007/s11111-026-00518-w)
Supplement: Supplementary file 1 — Supplementary Material [file 11111_2026_518_MOESM1_ESM.docx]

**Appendix 1.** Further information on the advantages and disadvantages of using objective and subjective wellbeing in policy

The “ECW” framework is designed to support sustainable development research and policy to holistically improve individual and collective wellbeing. By incorporating multidisciplinary concepts, the framework can overcome the limitations of single approaches (Addison et al., 2008) and address concerns that “by prioritising [OWB], other dimensions of wellbeing can be ignored or damaged” (Copestake, 2008; p.591).

Single, quantifiable OWB measures (i.e., GDP/capita) are often preferred due to greater data availability and simpler interpretation (Laderchi et al., 2003). However, unidimensional OWB measures create a narrow focus where policymakers interpret progression from a capitalist perspective (White, 2010). In contrast, multidimensional OWB (i.e., basic needs deprivation) (Alkire & Foster, 2011) may “help identify the weakest thread in the tapestry of human well-being” (Agarwala et al., 2014; p.441), and allow policy to target different causes and solutions to varying challenges. However, some criticise “basic needs” approaches for creating “false simplicity”. For instance, assuming building more schools improves wellbeing without acknowledging the broader structural and social changes required to enable communities to benefit from material improvements (Ghai, 1978).

Despite policy targets, including SDGs, commonly focusing on objective indicators, there is a growing recognition that “better outcomes may result from attempts to maximise happiness…rather than financial wealth” (Agarwala et al., 2014; p.438). SWB, including happiness, creates channels for public voices to be reflected in policy (Diener & Suh, 1997; Dolan & White, 2007), improves community buy-in and enhances policymaker accountability (De Schutter, 2021). Flik and Praag (1991) state “individuals themselves are the best judges of their own situation” (p.313); therefore, SWB measures can minimise the influence of external assumptions and provide more accurate, context-specific understandings (Nunan, 2015). Furthermore, individuals’ emotions and perceptions govern behaviour (Alam & Mallick, 2022). Therefore, exploring SWB can improve decision-makers’ understanding of communities’ policy responses, particularly if they do not align with targeted OWB outcomes (Coulthard, 2011; Gross-Camp, 2017).

Unlike unidimensional OWB measures, SWB captures wide-ranging information, including emotions towards health, future opportunities and housing (Mahmood et al., 2019; Marks, 2007; Reyes-García et al., 2016). However, the complex information can restrict policymakers from pinpointing specific causes/solutions (Brüggen et al., 2017). Furthermore, if policy only monitors success subjectively, it may result in the “grumbling rich man” being prioritised over the “contented peasant” (Sen, 1983; p.160). Defining objectively poor households as “content” can romanticise financial poverty (Kay & Jost, 2003), rationalise inequality and discourage material investments (Davis, 2014).

Therefore, sustainability policy should incorporate OWB *and* SWB to improve communities’ comprehensive wellbeing and overcome limitations associated with singular approaches. For example, a purely objective approach could omit information on how communities’ collective norms, a foundation of SWB, could be used to harness knowledge sharing and cooperative action to support environmental policies (Bouma et al., 2008). Certain measures combine OWB and SWB within a single indicator (i.e., Bhutan Gross National Happiness Index); however, this can raise various issues, including ethical dilemmas if prioritising material improvements over emotional needs (Rojas, 2011). Therefore, the “ECW” framework depicts OWB and SWB as interconnected, yet separate, concepts

**Appendix 2.** Summary of existing environment-wellbeing frameworks, including limitations and the benefits adopted by the new ECW framework. This review draws inspiration from Schleicher et al. (2018).

| **Framework** | **Millennium Ecosystem Assessment (MEA)** | **Environmental Endowments and Entitlements (EEE)** | |
| --- | --- | --- | --- |
| Overarching statement | MEA illustrates an interconnected system between indirect drivers (i.e., economic markets), direct drivers (i.e., climate change), ecosystem services and human wellbeing | EEE acknowledges how wellbeing differs due to varying endowments^[[1]](#footnote-1)^ and entitlements^[[2]](#footnote-2)^ to environmental resources, governed by multi-scale institutions | |
| Wellbeing definition | Basic material, health, social relations, security & freedom of choice; defined by “voices of the poor” research (Narayan & Petesch, 2002) | Endowments and entitlements shape individuals’ capabilities. Endowments alone are insufficient in achieving wellbeing | |
| Role of environment | Determinant (potential component) | Determinant | |
| Environment-wellbeing relationship | Ecosystem services are split into four groups to highlight the different mechanisms in which they can support multidimensional wellbeing (provisioning, regulating, cultural, supporting) | “Environmental goods” are accessed and negotiated to achieve wellbeing outcomes | |
| Scale | Macro-scale; global challenges | Macro, meso and micro-scales | |
| Limitations | Macro-scale focus oversimplifies the different climatic impacts faced within LMIC localities, and may overlook social differentiation (Nunan, 2015)  No interaction between “Indirect drivers” and “Ecosystem services” means differences in natural resource access, which influence if actors can transfer ecosystem services into wellbeing outcomes, are overlooked (Daw et al., 2011)  No feedback between “Wellbeing & poverty reduction” and “Ecosystem services” also ignores how low levels of wellbeing could influence ecosystem service quality and availability (Jehan & Umana, 2003) | EEE views “environmental goods” from a positive perspective, potentially overlooking how climate hazards can negatively impact households’ capabilities and wellbeing | |
| Benefits (adopted by “ECW”) | Acknowledges the macro-scale drivers which can influence wellbeing outcomes and environmental associations | Acknowledges the role of cross-scale institutions in governing households’ endowments and entitlements (incorporated in the “relational context”)  Supports the use of qualitative research to explore how social differentiation and inequality influence environment-wellbeing relationships | |
| Key references | Daw et al., 2011; Jehan & Umana, 2003; MEA, 2005 | Gasper, 2007; Leach et al., 1999; Sen, 1999 | |
| **Framework** | **Economics of Ecosystems and Biodiversity (TEEB)** | **Driver–Pressure–State–Welfare–Response (DPSWR)** |  |
| Overarching statement | TEEB aims to draw attention to the invisibility of nature in the economic choices we make by quantifying the economic value of ecosystem services | DPSWR is a tool to identify the causes of, and manage, environmental issues. The challenges and outcomes function at multiple scales within an integrated socio-ecological system |  |
| Wellbeing definition | Economic (GDP), socio-cultural (spiritual benefits) and biophysical (resilience) | Welfare (human wellbeing) & State (ecosystem wellbeing) |  |
| Role of environment | Component and determinant | Determinant |  |
| Environment-wellbeing relationship | Environmental concepts incorporated within direct drivers (i.e., land-use change) and external drivers (i.e., climate change)  TEEB separates ecosystem processes and services, with ecosystem services benefitting different strands of human wellbeing; which can be quantified to provide a monetary value | Driver (broad-scale development in a social system which aims to improve human welfare) creates Pressure (direct actions of humans on environment), which impacts the State (ecosystem wellbeing) and Welfare (human wellbeing). A change in Welfare leads to a Response (adaptation) which can mitigate the Driver, Pressure and State |  |
| Scale | Macro-scale; similar terminology to MEA | Socio-ecological system scale |  |
| Limitations | Difficulty in quantifying intangible cultural/spiritual benefits  Monetary valuations may not be contextually meaningful to subsistence communities  TEEB is unrealistically linear, overlooking potential feedback effects; for example, governance structures could influence all other components within the framework | Potentially oversimplified and overlooks certain complexities; for example, State and Welfare could be influenced by multiple different Drivers, Pressures and Responses  “Pressure” assumes a negative influence from human actions, potentially detracting from the focus on sustainable activities that maximise ecosystem and human wellbeing |  |
| Benefits (adopted by “ECW”) | Acknowledges the macro-scale direct and indirect drivers which can influence wellbeing outcomes and environmental associations  The role of institutions and governance influences how wellbeing is conceptualised and how it is impacted by environmental conditions | Acknowledges the macro-scale drivers which influence relationships between human and ecosystem wellbeing  Acknowledges individuals’ and communities’ agency to respond to environmental and wellbeing changes |  |
| Key references | Small et al., 2017; TEEB, 2022 | Cooper, 2013; Gari et al., 2015; Kelble et al., 2013; Rekolainen et al., 2003 |  |

| **Framework** | **Sustainable Livelihoods Framework (SLF)** | | | **Vulnerability framework** |
| --- | --- | --- | --- | --- |
| Overarching statement | SLF depicts livelihoods to function within various contexts (political, historical, socio-economic, climatic), which alongside overarching institutions and policy restraints, control access to different resources and livelihood outcomes  A ”livelihood” framework approach (Nunan, 2015) | | | The Vulnerability framework adapts SLF by incorporating wellbeing, rather than livelihood, outcomes, and specifically focuses on the environmental context (short-term climate shocks, long-term stresses and seasonality) |
| Wellbeing definition | Livelihood (working days, poverty, capabilities) & Sustainability (livelihood adaptation and sustainable natural resources) | | | Wellbeing outcomes dependent on resilience (health, risk aversion, community cohesion and environment) |
| Role of environment | Determinant (potential component) | | | Determinant (potential component) |
| Environment-wellbeing relationship | Natural capital listed as one of the key resources actors can use to develop their livelihood strategies and outcomes | | | Environmental shocks, stresses and seasonal shifts impact wellbeing outcomes depending on actors’ exposure, sensitivity and coping/adaptive practices |
| Scale | Multi-scale (macro-scale context, meso-scale institutions, micro-scale livelihoods) | | | Multi-scale; similar to SLF, yet with a specific “environmental” focus |
| Limitations | Potentially overlooks the underlying influence of power and entitlement upon wellbeing, and interactions between capital assets  Disproportionately focuses on objective “checklists”, and therefore fails to capture the meanings attached to livelihood resources  Focus on household-level livelihood strategies  Capitals only illustrated as “means” to wellbeing, rather than “ends” | | | Defines exposure through location and functional group, yet “functional group” arguably controls “sensitivity” to harm, rather than exposure. Different livelihood groups may all be exposed to a hazard; yet, the hazard only constitutes a “risk” if the community is vulnerable  SLF capitals illustrated to influence “sensitivity”, yet not “adaptive capacity” |
| Benefits (adopted by “ECW”) | Facilitates cross-scale analysis to understand different factors influencing livelihood outcomes  Flexibility to incorporate community-level capitals (Berchoux & Hutton, 2019)  Flexibility to incorporate elements from other frameworks (i.e., “legal capitals”, such as land rights, within “livelihood resources”) to address issues of social differentiation  Supports participatory methods to enable communities to define context-specific outcomes and historical influences  SLF capitals provide broad groupings to aid the quantification of environment-wellbeing relationships. Broad capitals can be flexibly interpreted depending on research aims and data access, ensuring key documented elements are not overlooked, and facilitating analytical comparability  Highlights the role of “livelihoods” within the environment-wellbeing system  Acknowledges actors’ agency to adapt to changing environmental conditions and wellbeing | | | Breakdown of the different temporal scales of environmental challenges (short-term shocks, longer-term stresses, and seasonal shifts)  Illustrates the potential for similar climate events to have differing effects based on exposure, sensitivity and response  Acknowledges individuals’ and communities’ agency to respond to environmental and wellbeing changes |
| Key references | Scoones, 1998 | | | Scott, 2006 |
| **Framework** | | **IPCC risk framework** | **Wellbeing in Developing Countries (WeD)** | |
| Overarching statement | | A climate hazard constitutes a risk depending on the community’s vulnerability (exposure, sensitivity, adaptive capacity) | WeD focuses on households’ rights to resources, and the meanings attached to such resources. Wellbeing is constructed by the interaction of material, relational and subjective elements  Wellbeing is a process created within time and space  A ”wellbeing” framework approach (Nunan, 2015) | |
| Wellbeing definition | | “Wellbeing” dimensions as *means* to controlling vulnerability and *end outcomes* (environmental, social, economic) | Material, Relational & Subjective, constructed in time and space  *Etic* (objective) and *Emic* (subjective) components, encountered through individual and collective experiences | |
| Role of environment | | Determinant (potential component) | Component (potential determinant) | |
| Environment-wellbeing relationship | | Wellbeing outcomes are determined by exposure to climate hazards, sensitivity (the likelihood of experiencing harm) and adaptive capacity (the ability for actors to adjust, or take advantage, of changing conditions)  Vulnerability is situation-specific, meaning a community may be vulnerable to one hazard, yet not another, depending upon their livelihood characteristics and resources  Vulnerability can also vary over time, or communities may possess underlying vulnerability which is unveiled following repeat hazards | Gough & McGregor’s (2007) WeD framework does not explicitly incorporate “environment”, whereas White’s (2010) adapted framework includes environmental resources within “social wellbeing” | |
| Scale | | Vulnerability is heterogeneous across various scales (i.e., landscape or intrahousehold) | Bridges the gap between different scales by distinguishing between universally recognised “needs” and locally-formed, cultural “wants” | |
| Limitations | | Overly technical and positivist, which can restrict research on values and risk perceptions within social sciences  Interpretation of risk arguably does not account for governance or political influences upon resource access and wellbeing outcomes (Tangney, 2020)  Changing definitions (i.e., combining adaptive capacity and sensitivity within the IPCC 5^th^ Assessment Report) have potentially limited the framework’s adoption (Estoque et al, 2023) | Encapsulating “environment” within “social wellbeing” arguably narrows the focus on how “environment” can influence multiple strands of human wellbeing. Limiting the capacity for the framework to be used in interdisciplinary research with natural sciences (Agarwala et al., 2014)  Incorporating SWB as an equal partner alongside OWB is arguably unjust, especially when target communities have immediate material needs  WeD potentially accepts the role of existing collective values/goals within the “relational context”, rather than challenging the underlying power structures which create social norms  White’s (2010) adaptation presents “relational” wellbeing as a third component, alongside “subjective” and “material”. Yet, it could be argued that “relational” is a mediating factor influencing OWB, SWB and their relationship | |
| **Framework** | | **IPCC risk framework (continued…)** | **Wellbeing in Developing Countries (WeD) (continued…)** | |
| Limitations (continued…) | |  | Wellbeing is defined as a process, suggesting it is not also an outcome which can be measured and monitored at a given point | |
| Benefits (adopted by “ECW”) | | Forms the basis of the “ECW” framework in interpreting OWB & SWB as “means” to further wellbeing by influencing vulnerability (adaptive capacity & sensitivity)  Differentiation between “sensitivity” and “adaptive capacity” within overarching “vulnerability”  Acknowledges spatial differences and the contextual controls which can mean hazards do not constitute “risks” in all situations  Clarifies how “Exposure” is a key component, yet not a determinant, of “risk” | Acknowledges wellbeing to incorporate OWB & SWB components  Recognises the need for integrated natural and social sciences when understanding environment-wellbeing systems (Nuijten, 2011)  Relational context prevents wellbeing from being exclusively viewed as individualistic, as collective expectations and beliefs also influence wellbeing. Also, the relational lens highlights how wellbeing is conceptualised and experienced can change over time  The “relational” focus supports small-scale studies, which can unveil local nuances which macro-scale frameworks may miss (Coulthard, 2011) | |
| Key references | | Adger, 2002; Cardona et al., 2012, Dasgupta & Baschieri, 2010; IPCC, 2024; Thomas et al., 2019 | Gough & McGregor, 2007; White, 2010 | |

**Appendix 3.** Supporting diagrams of the selected environment-wellbeing frameworks presented in Appendix (2)


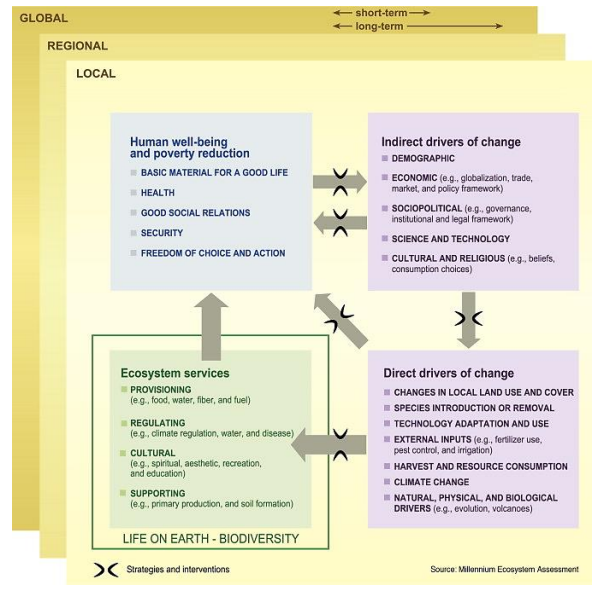


Millennium Ecosystem Assessment (MEA) framework. Sourced from Fisher et al. (2013, p.1103) and MEA (2005)


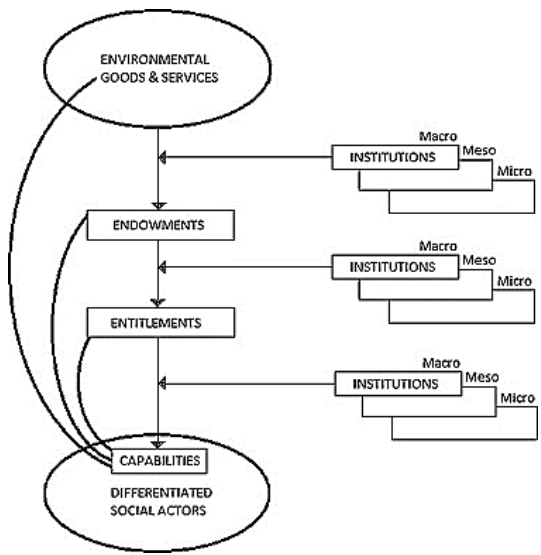


Environmental Endowments and Entitlements (EEE) framework. Sourced from Fisher et al. (2013, p.1101) and Leach et al. (1999)


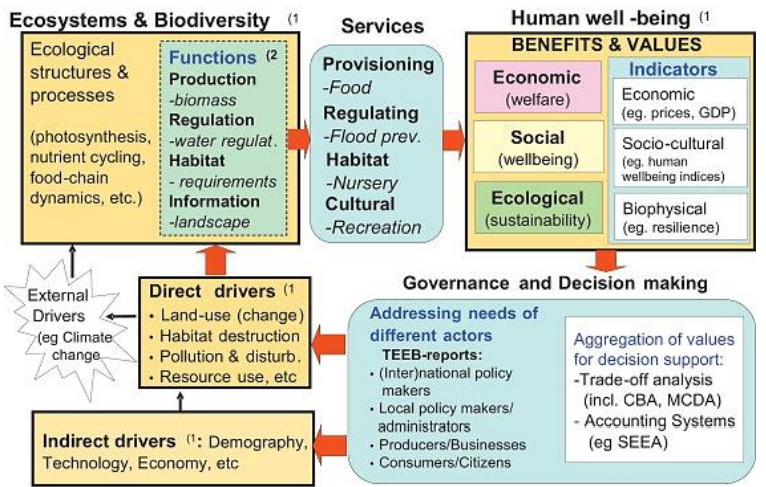


The Economics of Ecosystems and Biodiversity (TEEB) framework. Sourced from Fisher et al. (2013, p.1106) and TEEB (2010)


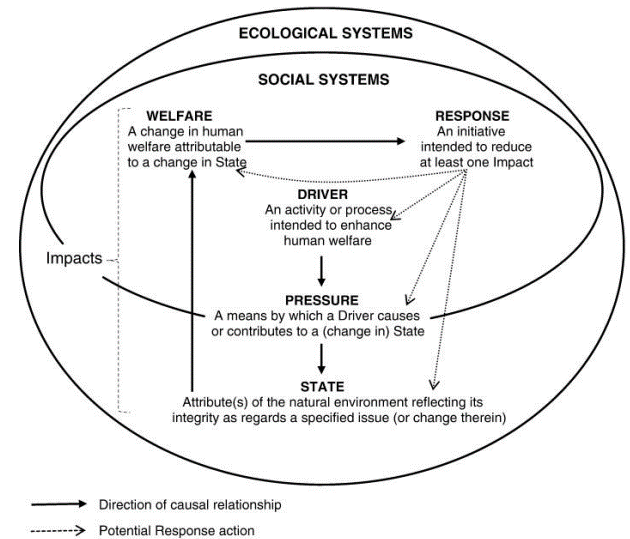


The Driver–Pressure–State–Welfare–Response (DPSWR) framework. Sourced from Cooper (2013, p.111)

The Sustainable Livelihoods Framework (SLF). Sourced from Scoones (1998, p.4)


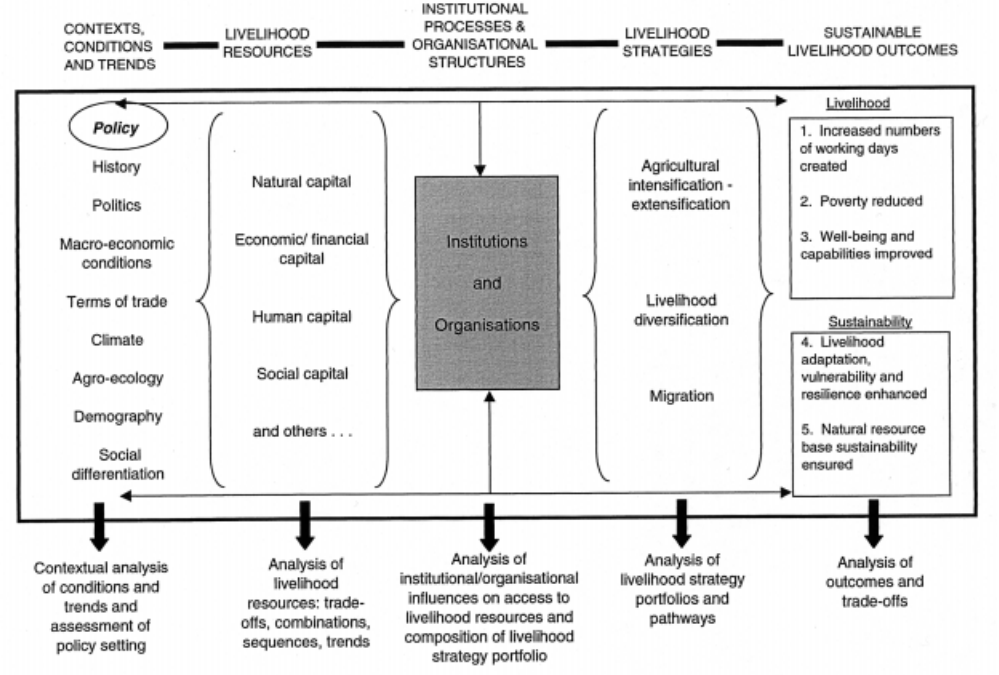


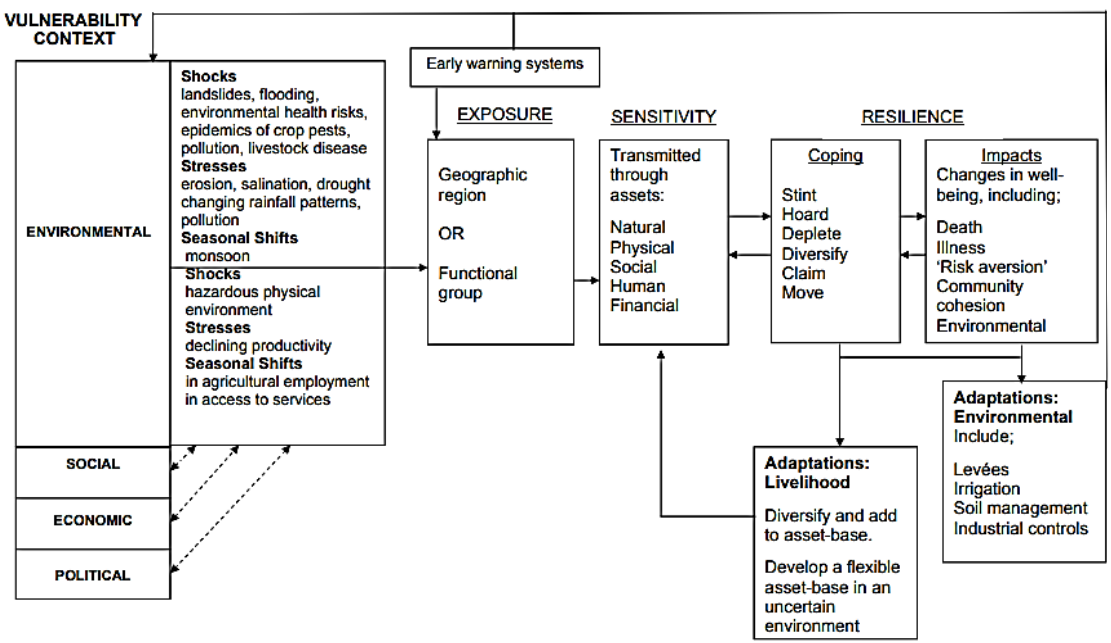


The Vulnerability Framework, an adaptation of SLF. Sourced from Scott (2006, p.5)


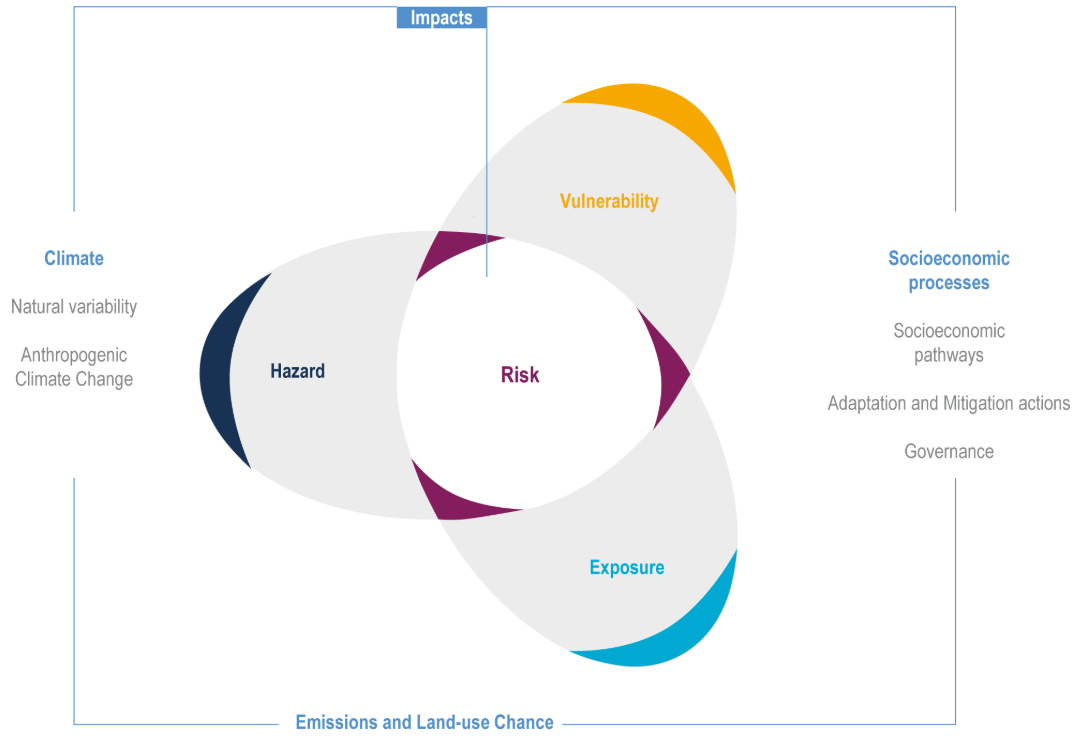
The IPCC Risk framework (Begum et al., 2022), developed from the IPCC Special Report on Managing the Risks of Extreme Events and Disasters to Advance Climate Change Adaptation and IPCC Fifth Assessment Report. Minor amendments were made in the Sixth Assessment Report which explicitly acknowledges how adaptive responses can moderate different elements of risk.


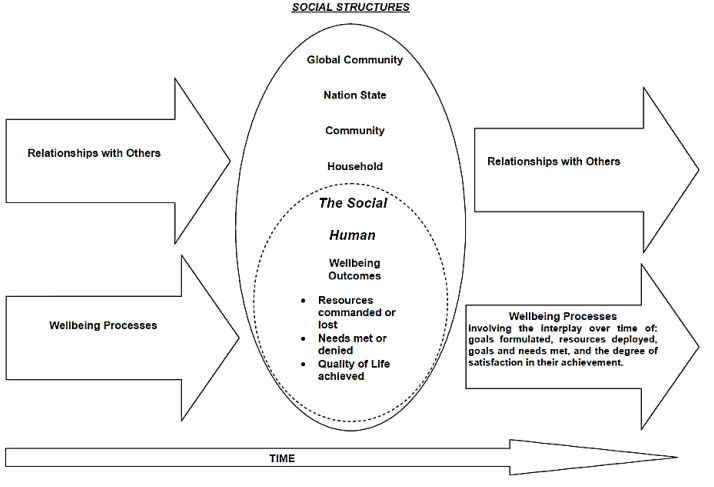


The Wellbeing in Developing Countries framework (WeD). Sourced from Gough & McGregor (2007, p.34)


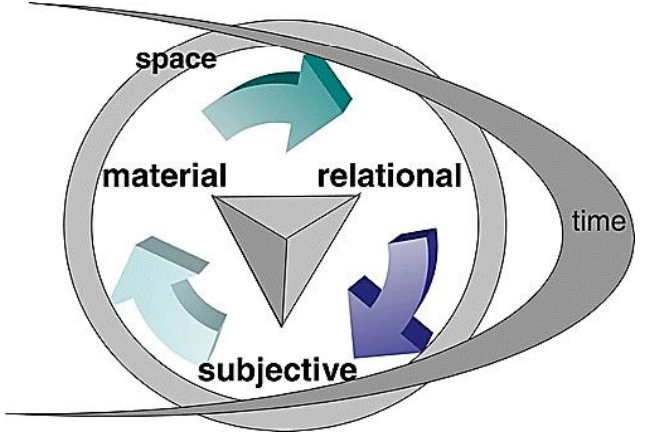


A dynamic visual highlighting how the Wellbeing in Developing Countries framework (WeD) incorporates material (objective) and subjective components, which are influenced by the relational context, space and time in which wellbeing is constructed. Sourced from White (2010, p.165)


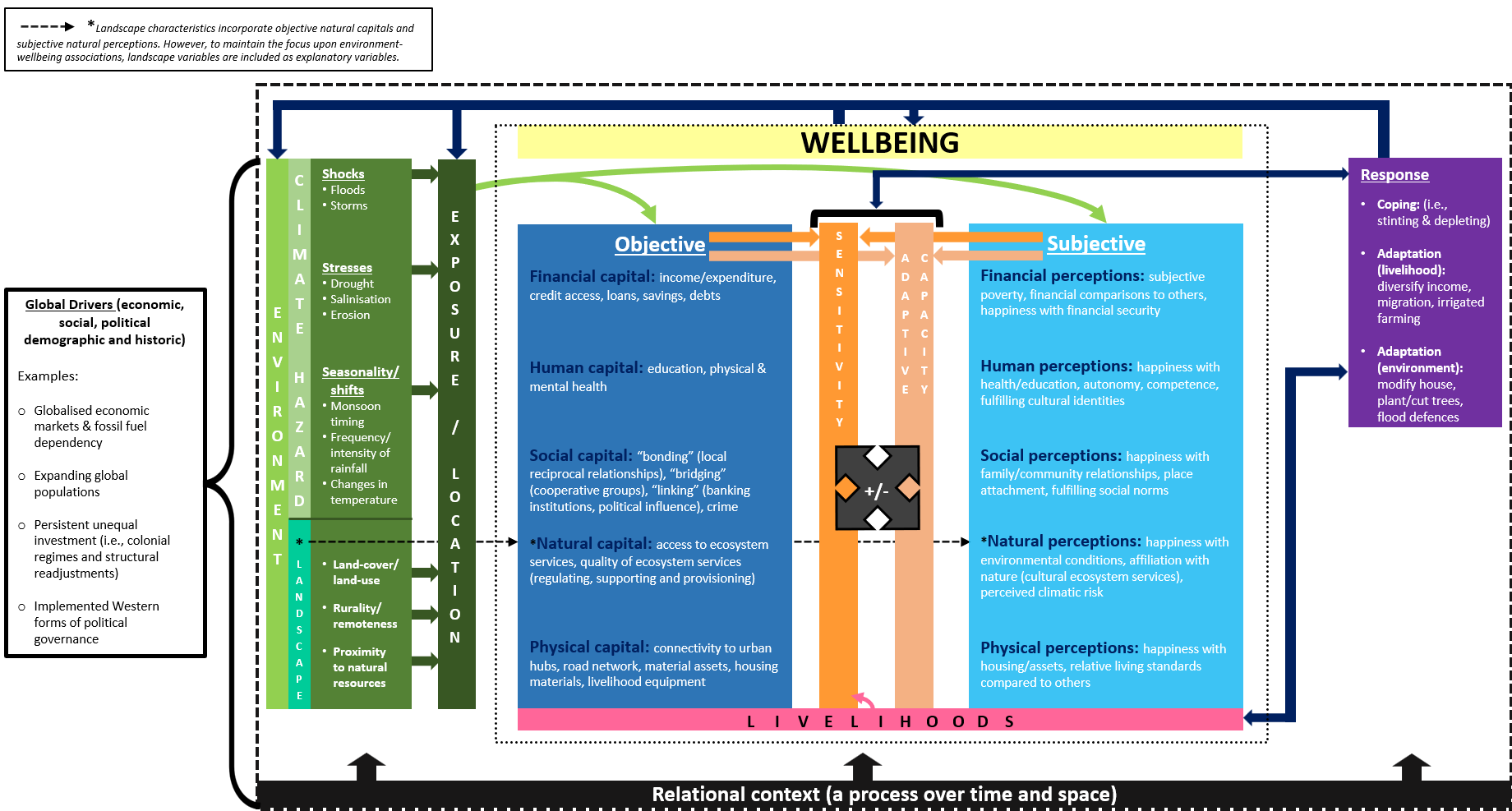
**Appendix 4.** The Environment and Comprehensive Wellbeing (ECW) conceptual framework – without the reference numbers cited in the main text

| **Model 1 (expenditure poverty) Variable** | **Odds coefficient**  **[exp(β)] (S.E)** |
| --- | --- |
| **Climatic shocks, stresses & shifts** | |
| **Environmental impact from drought (ref: No impact)** |  |
| Impacted | *0.660 (0.282)* |
| **Economic impact from flooding (ref: No impact)** |  |
| Impacted | 0.664 (0.138)** |
| **Environmental landscape/remoteness** | |
| **Travel time to Accra (ref: 1-2 hours)** |  |
| 2-3 hours | 1.482 (0.345)* |
| 3+ hours | 2.046 (0.456)*** |
| **Distance to inland water (km)** |  |
| Distance (km) | *1.090 (0.069)* |
| **River vegetation in 2km community buffer (ref: No)** |  |
| Yes | 0.236 (0.104)*** |
| **Savannah grassland in 2km community buffer (%)** |  |
| Percentage grassland coverage | 1.026 (0.007)*** |
| **Mean 2016 EVI value for 2km community buffer (ref: Low)** |  |
| Medium EVI | *0.636 (0.239)* |
| High EVI | *5.006 (2.901)**** |
| **Physical assets** | |
| **Drinking water source (ref: Piped or tubewell)** |  |
| Dug well or open-source | *1.282 (0.367)* |
| **Household characteristics** | |
| **Household size** |  |
| 2-3 people | 7.522 (2.491)*** |
| 4-5 people | 27.565 (9.314)*** |
| 6-7 people | 49.957 (18.285)*** |
| 8+ people | 108.892 (45.109)*** |
| **% females in the household** |  |
| Percentage in household | 0.992 (0.003)*** |
| **Household livelihood cluster (ref: Salaried employee/business owner)** |  |
| Crop farmer | 2.185 (0.545)*** |
| Fisher/trade/transport/construction | 1.852 (0.417)*** |
| **Household head characteristics** | |
| **Highest education level (ref: No schooling)** |  |
| Primary education (below-basic) | 0.574 (0.112)*** |
| Lower secondary education (basic) | 0.649 (0.129)** |
| Higher secondary or higher education (above-basic) | 0.264 (0.066)*** |
| **Subjective characteristics** | |
| **Place/community attachment (ref: Low attachment)** |  |
| Medium attachment | *0.394 (0.115)**** |
| High attachment | *0.215 (0.065)**** |
| **Adaptation** | |
| **Current migrant outside household (ref: No)** |  |
| Yes | 1.453 (0.233)** |
| **Any form of adaptation in the last 5 years (ref: No)** |  |
| Yes | *0.628 (0.171)** |
| **Interactions** | |
| **Drinking water source x distance to inland water (km)** |  |
| Dug well or open-source x distance (km) | 1.271 (0.100)*** |
| **Mean 2016 EVI value x Household Adaptation** |  |
| Medium EVI x Adaptation [Yes] | 1.539 (0.612) |
| High EVI x Adaptation [Yes] | 0.152 (0.083)*** |
| **Environmental drought impact x place/community attachment** |  |
| Impacted x Medium attachment | 1.816 (0.869) |
| Impacted x High attachment | 3.582 (1.695)*** |
| **Additional model information** | |
| Intercept | 0.063 (0.033)*** |
| No. observations | 1,265 |
| Log Likelihood | -584.742 |

**Appendix 5.** The eight binary logistic regression models exploring OWB, SWB and aligning/opposing OWB-SWB. Models produced using the DECCMA survey dataset and supporting remote sensing datasets

| **Model 2 (basic needs deprivation) Variable** | **Odds coefficient**  **[exp(β)] (S.E)** |
| --- | --- |
| **Climatic shocks, stresses & shifts** | |
| **Environmental impact from drought (ref: No impact)** |  |
| Impacted | 0.443 (0.097)*** |
| **Exposure to drought (ref: Not exposed)** |  |
| Exposed | 2.659 (0.568)*** |
| **Self-reported change in rainfall in the last 5 years (ref: Changed)** |  |
| Stayed the same | 1.859 (0.555)** |
| **Environmental landscape/remoteness** | |
| **Distance from road intersect (ref: Closest to intersect)** |  |
| Group 2 (of 3) | 0.980 (0.197) |
| Group 3 (of 3, furthest from intersect) | 1.631 (0.322)** |
| **Travel time to district capital (ref: Group 4/4 [longest travel time])** |  |
| Group 1: Shortest travel time | 0.255 (0.057)*** |
| Group 2 | 0.587 (0.118)*** |
| Group 3 | 0.383 (0.081)*** |
| **Region (ref: Greater Accra)** |  |
| Volta | *0.289 (0.645)**** |
| **Built-up land in 2km community buffer (ref: None/Low coverage)** |  |
| Medium coverage | 1.204 (0.277) |
| High coverage | 0.538 (0.143)** |
| **Household characteristics** | |
| **Household religion (ref: Christian)** |  |
| Non-Christian | 2.119 (0.342)*** |
| **Household livelihood cluster (ref: Salaried employee/business owner)** |  |
| Crop farmer | 1.950 (0.432)*** |
| Fisher/trade/transport/construction | 1.700 (0.336)*** |
| **Household head characteristics** | |
| **Sex (ref: Male)** |  |
| Female | 1.660 (0.254)*** |
| **Age quartile (ref: Youngest quartile)** |  |
| Young quartile | 0.774 (0.153) |
| Medium quartile | 0.627 (0.131)** |
| Oldest quartile | 1.106 (0.246) |
| **Marital status (ref: Previously married – separated/widowed)** |  |
| Married/cohabitating | 0.794 (0.143) |
| Never married | 0.410 (0.114)*** |
| **Personality group (ref: Low personality)** |  |
| Medium personality | 1.025 (0.217) |
| High personality | 0.538 (0.143)** |
| **Adaptation** | |
| **Past migrant in household (ref: No)** |  |
| Yes | *0.387 (0.091)**** |
| **Any form of adaptation in the last 5 years (ref: No)** |  |
| Yes | 0.431 (0.071)*** |
| **Interactions** | |
| **Past migrant x Region** |  |
| Past migrant in household [Yes] x Volta | 2.647 (0.757)*** |
| **Additional model information** | |
| Intercept | 5.072 (2.187)*** |
| No. observations | 1,273 |
| Log Likelihood | -688.812 |

| **Model 3 (financial stress) Variable** | **Odds coefficient**  **[exp(β)] (S.E)** |
| --- | --- |
| **Climatic shocks, stresses & shifts** | |
| **Economic impact from storms (ref: No impact)** |  |
| Impacted | 2.553 (0.906)*** |
| **Exposed to storms (ref: Not exposed)** |  |
| Exposed | *0.432 (0.140)**** |
| **Self-reported change in rainfall in the last 5 years (ref: Changed)** |  |
| Stayed the same | 0.445 (0.137)*** |
| **Self-reported change in temperature in the last 5 years (ref: Changed)** |  |
| Stayed the same | 0.363 (0.133)*** |
| **Environmental landscape/remoteness** | |
| **Distance from road intersect (ref: Closest to intersect)** |  |
| Group 2 (of 3) | *0.936 (0.681)* |
| Group 3 (of 3, furthest from intersect) | *1.729 (1.562))* |
| **Travel time to Accra (ref: 1-2 hours)** |  |
| 2-3 hours | 1.924 (0.587)** |
| 3+ hours | 0.841 (0.216) |
| **Marshland in 2km community buffer (ref: No)** |  |
| Yes | 2.388 (0.549)*** |
| **Savannah grassland in 2km community buffer (ref: None)** |  |
| Low/medium coverage | 0.508 (0.111)*** |
| High coverage | 0.409 (0.131)*** |
| **Built-up land in 2km community buffer (ref: None/Low coverage)** |  |
| Medium/high coverage | *0.371 (0.107)**** |
| **Household characteristics** | |
| **Household religion (ref: Christian)** |  |
| Non-Christian | 0.581 (0.128)** |
| **Child/adult dependency ratio** |  |
| Child/adult ratio | 1.538 (0.234)*** |
| **Household livelihood cluster (ref: Salaried employee/business owner)** |  |
| Crop farmer | 2.449 (0.730)*** |
| Fisher/trade/transport/construction | 2.068 (0.488)*** |
| **Household head characteristics** | |
| **Highest education level (ref: No schooling)** |  |
| Primary education (below-basic) | 1.051 (0.297) |
| Lower secondary education (basic) | 0.623 (0.176)* |
| Higher secondary or higher education (above-basic) | 0.550 (0.165)** |
| **Employment status (ref: Permanent employment)** |  |
| Non-Permanent | 2.124 (0.588)*** |
| Dependant | 1.165 (0.381) |
| **Age quartile (ref: Youngest quartile)** |  |
| Young quartile | 2.007 (0.519)*** |
| Medium quartile | 2.235 (0.601)*** |
| Oldest quartile | 1.566 (0.462) |
| **Subjective characteristics** | |
| **Personality group (ref: Low personality)** |  |
| Medium personality | *0.936 (0.681)* |
| High personality | *0.677 (1.563)* |
| **Adaptation** | |
| **Current migrant outside household (ref: No)** |  |
| Yes | 0.499 (0.109)*** |
| **Interactions** | |
| **Distance from road intersect x Household head personality cluster** |  |
| Medium distance x Medium personality | 1.098 (0.865) |
| Medium distance x High personality | 0.761 (0.593) |
| Furthest distance x Medium personality | 1.533 (1.496) |
| Furthest distance x High personality | 0.293 (0.275) |
| **Built-up land cover x Exposure to storms** |  |
| Medium/high coverage x Exposed to storms | 6.565 (3.026)*** |
| **Additional model information** | |
| Intercept | 9.741 (6.001)*** |
| No. observations | 1,266 |
| Log Likelihood | -415.303 |

| **Model 4 (unhappiness) Variable** | **Odds coefficient  [exp(β)] (S.E)** |
| --- | --- |
| **Climatic shocks, stresses & shifts** | |
| **Environmental impact from salinity (ref: No impact)** |  |
| Impacted | 1.659 (0.341)** |
| **Environmental impact from storms (ref: No impact)** |  |
| Impacted | *0.769 (0.309)* |
| **Environmental landscape/remoteness** | |
| **Distance from major road (ref: Closest to major road)** |  |
| Group 2 (of 3) | 1.991 (0.459)*** |
| Group 3 (of 3, furthest from major road) | 1.008 (0.346) |
| **Cropland in 2km community buffer (%)** |  |
| Percentage coverage | *1.009 (0.006)* |
| **Wetland in 2km community buffer (ref: No)** |  |
| Yes | 1.880 (0.439)*** |
| **Subjective characteristics** | |
| **Place/community attachment (ref: Low attachment)** |  |
| Medium attachment | *0.960 (0.344)* |
| High attachment | *0.854 (0.312)* |
| **Personality group (ref: Low personality)** |  |
| Medium personality | *0.606 (0.181)** |
| High personality | *0.219 (0.074)**** |
| **Physical assets** | |
| **Household roof material (ref: Secure, non-natural materials)** |  |
| Non-secure, natural materials | 1.749 (0.332)*** |
| **Household characteristics** | |
| **Child/adult dependency ratio group (ref: Group 1 [lowest])** |  |
| Group 2 - medium | 1.415 (0.283)* |
| Group 3 - highest ratio | 2.277 (0.625)*** |
| **Household head characteristics** | |
| **Employment status (ref: Permanent)** |  |
| Non-Permanent | 1.539 (0.323)** |
| Dependant | 1.701 (0.496)* |
| **Adaptation** | |
| **Past migrant in household (ref: No)** |  |
| Yes | 1.450 (0.257)** |
| **Interactions** | |
| **Cropland community coverage x Place/community attachment** |  |
| % coverage x Medium attachment | 0.987 (0.007)* |
| % coverage x High attachment | 0.976 (0.008)*** |
| **Environmental storm impact x Household head personality group** |  |
| Impacted by storms x Medium personality | 2.071 (0.967) |
| Impacted by storms x High personality | 3.588 (1.845)** |
| **Additional model information** | |
| Intercept | 0.082 (0.039)*** |
| No. observations | 1,231 |
| Log Likelihood | -439.494 |

| **Model 5 (non-poor & happy) Variable** | **Odds coefficient**  **[exp(β)] (S.E)** |
| --- | --- |
| **Climatic shocks, stresses & shifts** | |
| **Environmental impact from drought (ref: No impact)** |  |
| Impacted | 0.564 (0.127)** |
| **Environmental impact from salinity (ref: No impact)** |  |
| Impacted | 0.702 (0.123)** |
| **Environmental impact from erosion (ref: No impact)** |  |
| Impacted | 0.697 (0.126)** |
| **Exposure to drought (ref: Not exposed)** |  |
| Exposed | 1.709 (0.386)** |
| **Self-reported change in rainfall in the last 5 years (ref: Changed)** |  |
| Stayed the same | 2.278 (0.720)*** |
| **Environmental landscape/remoteness** | |
| **Distance from major road (ref: Closest to major road)** |  |
| Group 2 (of 3) | 0.206 (0.055)*** |
| Group 3 (of 3, furthest from major road) | 1.309 (0.306) |
| **Mean 2016 EVI value for 2km community buffer (ref: Lowest)** |  |
| Low EVI | 1.260 (0.261) |
| Medium EVI | 1.298 (0.298) |
| High EVI | 0.477 (0.132)*** |
| **Change in built-up landcover % (2001-15) (ref: Stayed the same)** |  |
| Decreased | 1.531 (0.260)** |
| Increased | 2.012 (0.657)** |
| **Subjective characteristics** | |
| **Place/community attachment (ref: Low attachment)** |  |
| Medium attachment | 1.958 (0.463)*** |
| High attachment | 2.302 (0.547)*** |
| **Personality group (ref: Low personality)** |  |
| Medium personality | 1.308 (0.297) |
| High personality | 2.344 (0.542)*** |
| **Physical assets** | |
| **Drinking water source (ref: Piped or tubewell)** |  |
| Dug well or open-source | 0.462 (0.082)*** |
| **Household characteristics** | |
| **Household religion (ref: Christian)** |  |
| Non-Christian | 0.615 (0.101)*** |
| **Household size (ref: 1-person household)** |  |
| 2-3 people | 0.322 (0.078)*** |
| 4-5 people | 0.109 (0.027)*** |
| 6-7 people | 0.072 (0.020)*** |
| 8+ people | 0.039 (0.013)*** |
| **Household livelihood cluster (ref: Salaried employee/business owner)** |  |
| Crop farmer | 0.458 (0.106)*** |
| Fisher/trade/transport/construction | 0.622 (0.127)** |
| **Additional model information** | |
| Intercept | 4.207 (0.123)** |
| No. observations | 1,181 |
| Log Likelihood | -614.158 |

| **Model 6 (poor & happy) Variable** | **Odds coefficient  [exp(β)] (S.E)** |
| --- | --- |
| **Environmental landscape/remoteness** | |
| **Distance from major road (ref: Closest to major road)** |  |
| Furthest from major road | *5.888 (2.595)**** |
| **Travel time to district capital (ref: Group 4/4 [longest travel time])** |  |
| Group 1: Shortest travel time | 1.011 (0.383) |
| Group 2 | 0.474 (0.175)** |
| Group 3 | 1.507 (0.517) |
| **Cropland in 2km community buffer (ref: None)** |  |
| Low coverage | 0.212 (0.104)*** |
| Medium coverage | 0.353 (0.181)** |
| High coverage | 0.228 (0.102)*** |
| **Subjective characteristics** | |
| **Place/community attachment (ref: Low attachment)** |  |
| Medium attachment | 0.387 (0.121)*** |
| High attachment | 0.235 (0.081)*** |
| **Personality group (ref: Low personality)** |  |
| Medium personality | 0.723 (0.221) |
| High personality | 0.278 (0.102)*** |
| **Physical assets** | |
| **Drinking water source (ref: Piped or tubewell)** |  |
| Dug well or open-source | 2.835 (0.813)*** |
| **Household roof material (ref: Secure, non-natural materials)** |  |
| Non-secure, natural materials | 1.760 (0.456)** |
| **Household characteristics** | |
| **Household size (ref: 1–4 person household)** |  |
| 5+ people | *3.581 (1.561)**** |
| **% females in the household (ref: 50% or lower)** |  |
| Over 50% females | 0.561 (0.141)** |
| **Child/adult dependency ratio group (ref: Group 1 [lowest])** |  |
| Group 2 - medium | 2.136 (0.586)*** |
| Group 3 - highest ratio | 2.952 (1.066)*** |
| **Household head characteristics** | |
| **Employment status (ref: Permanent)** |  |
| Non-Permanent | 1.857 (0.513)** |
| Dependant | 3.565 (1.328)*** |
| **Past migrant in household (ref: No)** |  |
| Yes | 1.994 (0.506)*** |
| **Interactions** | |
| **Major road distance x household size** |  |
| Furthest distance x 5+ people household size | 0.354 (0.181)** |
| **Additional model information** | |
| Intercept | 0.068 (0.039)*** |
| No. observations | 1,343 |
| Log Likelihood | -259.316 |

| **Model 7 (non-poor & unhappy) Variable** | **Odds coefficient  [exp(β)] (S.E)** |
| --- | --- |
| **Climatic shocks, stresses & shifts** | |
| **Environmental impact from salinity (ref: No impact)** |  |
| Impacted | 2.167 (0.531)*** |
| **Exposed to flooding (ref: Not exposed)** |  |
| Exposed | 1.999 (0.461)*** |
| **Environmental landscape/remoteness** | |
| **Cropland in 2km community buffer (ref: None)** |  |
| Low coverage | 0.454 (0.139)*** |
| Medium coverage | 0.866 (0.274) |
| High coverage | 0.446 (0.163)** |
| **River vegetation in 2km community buffer (ref: No)** |  |
| Yes | 3.861 (1.436)*** |
| **Wetland in 2km community buffer (ref: No)** |  |
| Yes | 2.210 (0.816)** |
| **Subjective characteristics** | |
| **Place/community attachment (ref: Low attachment)** |  |
| Medium attachment | 0.772 (0.220) |
| High attachment | 0.496 (0.144)** |
| **Household characteristics** | |
| **Household size (ref: 1–4 person household)** |  |
| 5+ people | 0.528 (0.132)*** |
| **Additional model information** | |
| Intercept | 0.067 (0.033)*** |
| No. observations | 1,275 |
| Log Likelihood | -313.102 |

| **Model 8 (poor & happy) Variable** | **Odds coefficient  [exp(β)] (S.E)** |
| --- | --- |
| **Climatic shocks, stresses & shifts** |  |
| **Economic impact from drought (ref: No impact)** |  |
| Impacted | 1.493 (0.261)** |
| **Economic impact from flooding (ref: No impact)** |  |
| Impacted | 0.563 (0.116)*** |
| **Environmental impact from erosion (ref: No impact)** |  |
| Impacted | 1.540 (0.271)** |
| **Environmental landscape/remoteness** |  |
| **Region (ref: Greater Accra)** |  |
| Volta | 3.628 (0.792)*** |
| **Cropland in 2km community buffer (ref: None)** |  |
| Low coverage | 3.293 (0.866)*** |
| Medium coverage | 2.488 (0.742)*** |
| High coverage | 2.956 (0.724)*** |
| **River vegetation in 2km community buffer (ref: No)** |  |
| Yes | 0.339 (0.113)*** |
| **Physical assets** |  |
| **Drinking water source (ref: Piped or tubewell)** |  |
| Dug well or open-source | 1.841 (0.326)*** |
| **Latrine facility (ref: Flushing)** |  |
| Pit/Public/KVIP (ventilated pit) | 2.107 (0.797)** |
| No facility | 3.319 (1.296)*** |
| **Household characteristics** |  |
| **Household size (ref: 1-person household)** |  |
| 2-3 people | 4.072 (1.268)*** |
| 4-5 people | 11.053 (3.398)*** |
| 6-7 people | 14.995 (4.926)*** |
| 8+ people | 32.438 (12.156)*** |
| **Household head characteristics** |  |
| **Highest education level (ref: No schooling)** |  |
| Primary education (below-basic) | 0.775 (0.143) |
| Lower secondary education (basic) | 0.854 (0.166) |
| Higher secondary or higher education (above-basic) | 0.376 (0.094)*** |
| **Adaptation** |  |
| **Current migrant outside household (ref: No)** |  |
| Yes | 1.471 (0.220)*** |
| **Additional model information** |  |
| Intercept | 0.003 (0.002)*** |
| No. observations | 1,237 |
| Log Likelihood | -600.313 |

**Appendix 6.** The methodology for the two OWB and two SWB binary wellbeing measures developed from the DECCMA survey dataset. Information on the construction of the aligning/opposing OWB-SWB measures also presented

Expenditure poverty (OWB)

Each household's recorded expenditure types were summed, excluding house rent and loan repayments. Renting costs were removed to avoid incorrectly identifying homeowners as experiencing poverty, and loan repayments were excluded as they insufficiently relate to living standards (Arndt & Tarp, 2016). Included expenditure types: food, household essentials (i.e., electricity), education, health, livelihood inputs (i.e., fertilisers), sporadic house costs (i.e., repairs), non-essential household costs (i.e., furniture), “Other” (i.e., weddings & funerals), insurance and supporting migrants in hardship.

Household expenditure was adjusted for regional living costs, and equivalised using the Ghanaian Statistical Service (GSS, 2018) calorie intake scale to provide “expenditure/equivalent adult per year”. Equivalisation facilitated comparison between households (Batana & Cockburn, 2018) by accounting for differences in household size, age composition and economies-of-scale (OECD, 2011).

Adjusted, equivalised household expenditure was compared to the GSS upper poverty line. The threshold (GH¢1,760.8 adult equivalent/year) is based on costings of essential food and non-food items, such as cleaning products and energy. Households with expenditure below the threshold were classified as experiencing poverty.

Basic needs deprivation (OWB)

The baseline basic needs deprivation measure was captured using Alkire & Foster’s (2011) “dual cut-off” count method. Twelve basic needs indicators (Table A6), adapted from existing basic needs studies (GSS, 2020; Santos & Villatoro, 2018; Streeten, 1984), were constructed from the DECCMA dataset. A “deprivation” threshold was applied for each indicator. For example, a household was deprived in “education” if all members aged 15+ had not completed a basic education. Following Alkire & Santos’ (2010) approach, “nested” weighting was applied to the indicators. Indicators were grouped by financial, human, social and physical capitals, as outlined in the Sustainable Livelihoods Framework (SLF) (Scoones, 1998), with each group weighted equally overall (Table A6).

Next, a second threshold defining the proportion of deprivations needed to be deprived “overall” was applied. A household was defined as deprived “overall” if it experienced at least 50% weighted deprivations, meaning the cumulative nested weight was equal to, or greater than, 6 (González et al., 2021; Hjelm et al., 2016). The 50% threshold was selected as it provided the most comparable result to GSS (2020) multiple deprivation estimates. It also fulfilled existing criteria for multiple deprivation classification, including the equivalent of two full “nested” deprivations, used in UNICEF Child Poverty reports (Alkire & Foster, 2011), and the requirement for the majority of deprivation components to be experienced (Aguilar & Sumner, 2020).

**Table A6** Individual deprivation indicators incorporated within the basic needs deprivation measure, grouped within the Sustainable Livelihoods Framework capitals (Scoones, 1998); produced using DECCMA data. “Nested” baseline weights also presented

| **Capital group** | **Basic need** | **Indicator** | **“Nested” baseline weight** |
| --- | --- | --- | --- |
| **Financial** | Employment | One or more household members are unemployed | 1 |
|  | Excess capital | Monthly expenditure on food >60% total expenditure | 1 |
|  | Bank access | No access to bank or loan service | 1 |
| **Human** | Education | All household members aged 15+ without basic education | 1.5 |
|  | Healthcare access | >5km from nearest hospital | 1.5 |
| **Social** | Cooperative membership | Not a member of a community cooperative network | 1.5 |
|  | Network size | Under 3 family/friends with migration experience | 1.5 |
| **Physical** | Roof quality | Low-quality roof material | 0.6 |
|  | Latrine | Unsafe latrine facility | 0.6 |
|  | Drinking water | Unsafe drinking water source | 0.6 |
|  | No overcrowding | Overcrowded household (< 9m^2^ house space per person) | 0.6 |
|  | Homeownership | Home is not owned | 0.6 |

Financial stress (SWB)

Measured using an adequacy approach (Mahmood et al., 2018). Where financial stress was defined by the response to “*do you feel under stress with your current income?* (**yes/no**)”.

Unhappiness (SWB)

Happiness was captured using a life domains approach, which assumes happiness with different components additively represents overall happiness (Rojas, 2006). This approach was selected over a “global” approach, which captures “general” happiness, as studies suggest life domains responses draw on different information and specific experiences, compared to “abstract” global evaluations (Cummins et al., 2003; Kozma et al., 2000; Lent, 2004; Schwarz & Strack, 1999). Therefore, “life domain” approaches are arguably more capable of capturing less-tangible wellbeing components, such as community satisfaction (Fagerholm et al., 2016).

Ordinal principal component analysis was used to agglomerate 8 correlating Likert scale measures of happiness, incorporating household heads’ happiness with; drinking water, food security, housing, economic security, health, community interactions, family interactions and environment (5-point Likert scale). Next, k-means clustering was used to formulate low, medium and high happiness clusters (Vyas & Kumaranayake, 2006). The “low” cluster was defined as “unhappy” in the binary outcome variable.

Aligning/opposing OWB-SWB

Four additional binary variables were constructed combining objective expenditure poverty and subjective unhappiness. These variables were selected to reflect opposite ends of the objective-subjective spectrum.

|  | **Life domains unhappiness** | |
| --- | --- | --- |
| **Expenditure poverty** | **No** | **Yes** |
| **No** | “Non-poor & Happy” | “Non-poor & Unhappy” |
| **Yes** | “Poor & Happy” | “Poor & Unhappy” |

**Appendix 7.** Details of focus group and interview participants from all eight study sites (numbered on the map). Age profiles for all community interviews, focus group members, and DPOs also presented


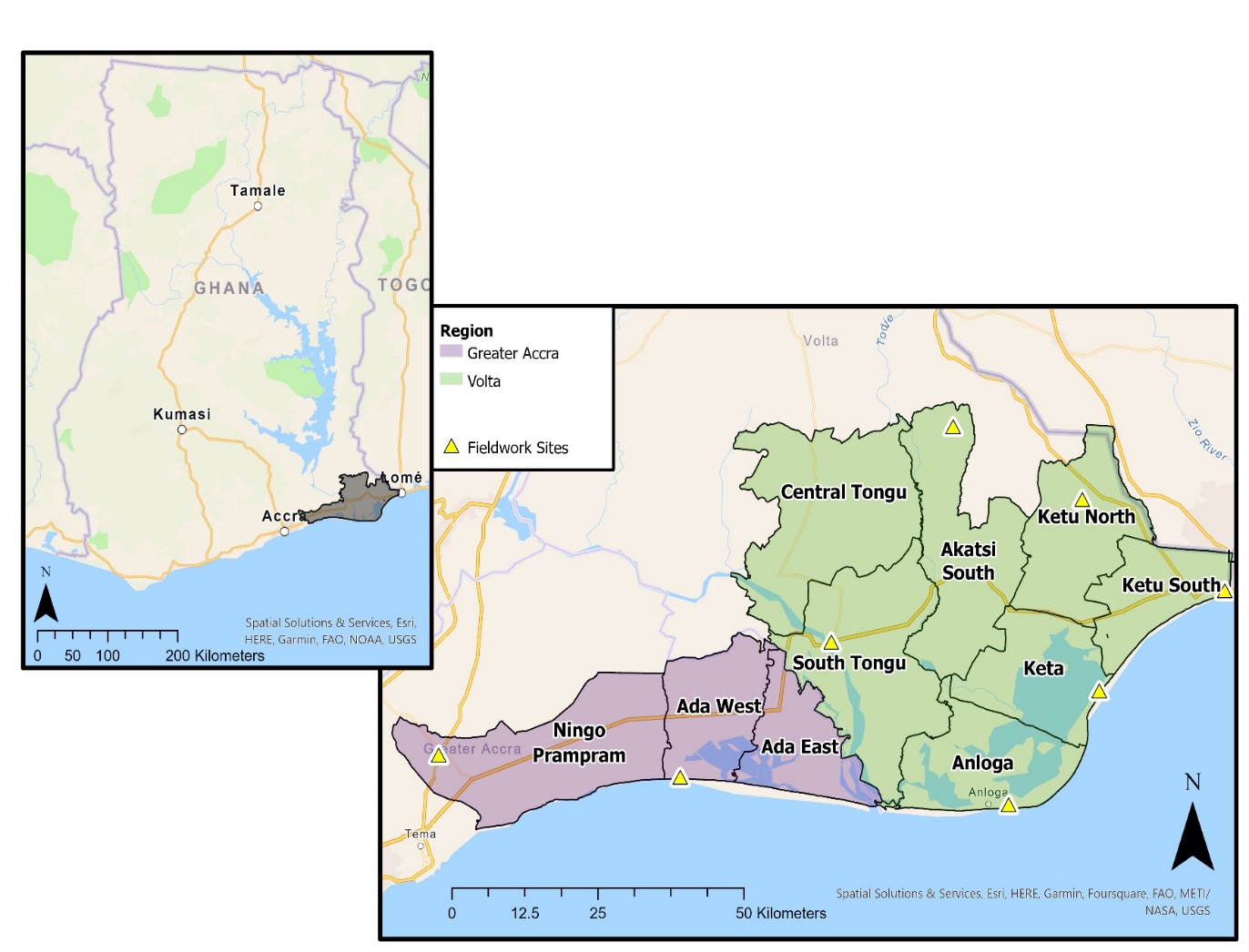


**1**

**2**

**3**

**4**

**5**

**6**

**7**

**8**

| **1) Afienya** | **Male FG** | **Female FG** | **Community interviews** | **DPO interview** |
| --- | --- | --- | --- | --- |
| No. participants | 7 | 6 | 1 (M) | 1 (F) |
| Duration | 01:45 | 01:38 | 00:33 | 00:55 |
| Age range | 32 - 49 | 21 - 55 | 32 – 41 | 42 - 51 |
| Language | English | Dangbe | Dangbe | English |
| Consent | Written | Written | Written | Written |

| **2) Anyamam** | **Male FG** | **Female FG** | **Community interviews** | **DPO interview** |
| --- | --- | --- | --- | --- |
| No. participants | 8 | 8 | 1 (M), 1 (F) | 1 (F) |
| Duration | 01:47 | 02:09 | 00:42 (M), 00:49 (F) | 00:59 |
| Age range | 28 - 48 | 25 - 62 | 32 – 41 (M & F) | 32 - 41 |
| Language | Dangbe | Dangbe | Dangbe | English |
| Consent | Written | Written | Written | Written |

| **3) Sogakope** | **Male FG** | **Female FG** | **Community interviews** | **DPO interview** |
| --- | --- | --- | --- | --- |
| No. participants | 6 | 10 | 1 (M), 1 (F) | 1 (M) |
| Duration | 01:32 | 01:49 | 00:32 (M), 00:32 (F) | 00:46 |
| Age range | 30 - 57 | 34 - 55 | 26 – 31 (M), 18 – 25 (F) | 32 - 41 |
| Language | Ewe | Ewe | English (M & F) | English |
| Consent | Written | Written | Written | Written |

| **4) Nyitawuta** | **Male FG** | **Female FG** | **Community interviews** | **DPO interview** |
| --- | --- | --- | --- | --- |
| No. participants | 8 | 8 | 1 (M), 1 (F) | 1 (M) |
| Duration | 02:08 | 01:42 | 00:46 (M), 00:47 (F) | 00:36 |
| Age range | 20 - 80 | 36 - 63 | x (M), 42 – 51 (F) | x |
| Language | Ewe | Ewe | Ewe | English |
| Consent | Written | Written | Written | Spoken |

| **5) Awlikope** | **Male FG** | **Female FG** | **Community interviews** | **DPO interview** |
| --- | --- | --- | --- | --- |
| No. participants | 8 | 8 | 1 (M), 1 (F) | 1 (M) |
| Duration | 01:51 | 01:52 | 00:58 (M), 01:03 (F) | 00:41 |
| Age range | 35 - 50 | 26 - 62 | 62 – 71 (M), 32 – 41 (F) | 42 - 51 |
| Language | Ewe | Ewe | English (M), Ewe (F) | English |
| Consent | Written | Written | Written | Written |

| **6) Aflao** | **Male FG** | **Female FG** | **Community interviews** | **DPO interview** |
| --- | --- | --- | --- | --- |
| No. participants | 7 | 8 | 1 (M), 1 (F) | 1 (M) |
| Duration | 01:04 | 01:27 | 00:37 (M), 00:39 (F) | 00:47 |
| Age range | 26 - 60 | 19 - 63 | 32 – 41 (M), 62 – 71 (F) | x |
| Language | Ewe | Ewe | Ewe (M), Ewe/Dangbe (F) | English |
| Consent | Written | Written | Written | Spoken |

| **7) Kedzi** | **Male FG** | **Female FG** | **Community interviews** | **DPO interview** |
| --- | --- | --- | --- | --- |
| No. participants | 9 | 7 | 2 (M) | 1 (M) |
| Duration | 01:51 | 01:45 | 00:45, 01:02 (M) | 00:38 |
| Age range | 32 - 66 | 38 - 58 | 32 – 41, 42 – 51 (M) | 52 - 61 |
| Language | Ewe | Ewe | 2 English (M) | English |
| Consent | Written | Written | Written | Written |

| **8) Anloga** | **Male FG** | **Female FG** | **Community interviews** | **DPO interview** |
| --- | --- | --- | --- | --- |
| No. participants | 10 | 8 | 1 (M), 1 (F) | 1 (M) |
| Duration | 01:30 | 01:40 | 00:48 (M), 00:37 (F) | 00:46 |
| Age range | 28 - 63 | 18 - 59 | 32 – 41 (M & F) | 32 – 41 |
| Language | Ewe | Ewe | English (M), Ewe (F) | English |
| Consent | Written | Written | Written | Written |


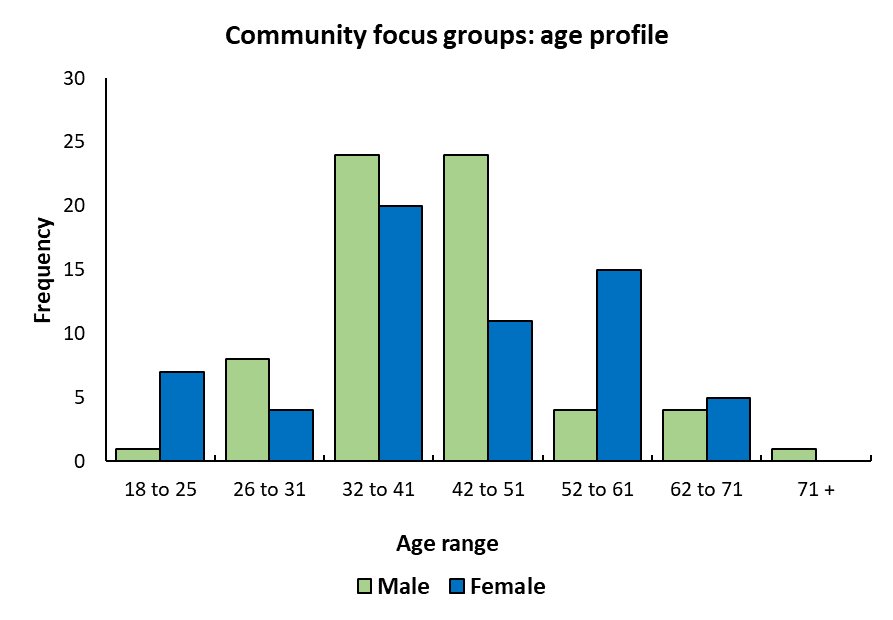


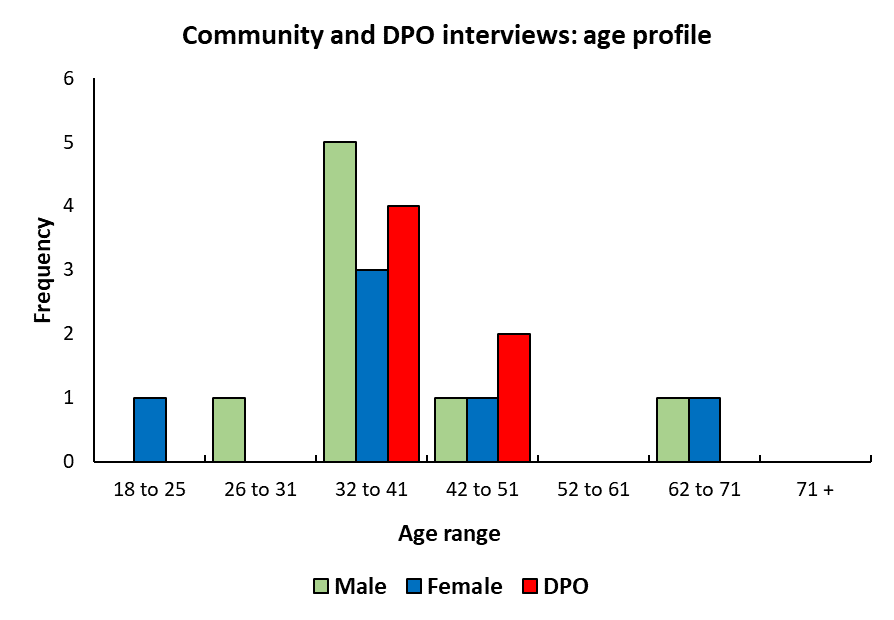


**Appendix 8.** Two causal loop examples from Anyamam (Ada West) and Kedzi (Keta) to illustrate the complex, multidirectional interconnections between the different elements of the “ECW” framework. Each numbered component is supported by mixed method findings. Focus group (FG), Community Interview (CI) and DPO interview, as well as gender (M/F) are recorded


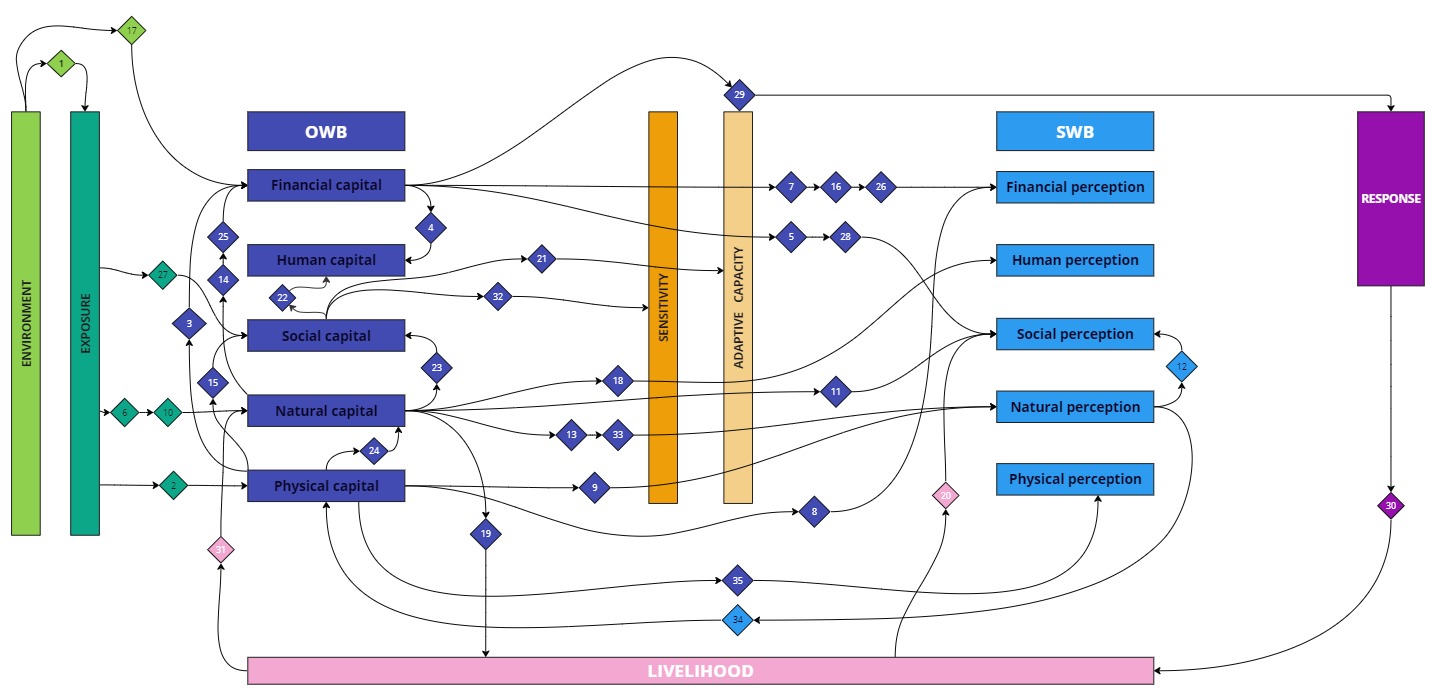


**Anyamam (Ada West)**

| **Appendix 8:** Anyamam supporting evidence | |
| --- | --- |
| **No.** | **Description** |
| 1 | The coastal location of Anyamam creates exposure to hazards such as flooding, salinisation and erosion; which are shown across various quantitative models to potentially cause negative OWB and SWB impacts; “*We have the sea here and there are times when the sea level rises and floods the community. This is another problem that we face*” (FG,F) |
| 2 | Hazards have destroyed housing and livelihood assets; “*when the sea is not happy, it flows into the town to pull down houses and as we speak, the sea has swallowed a lot of houses*” (FG,M) |
| 3 | Destruction/damage to livelihood assets, such as fishing boats, can restrict access to the financial capital received from trading natural resources; “*since the sea is our main source of fishing, so when the disaster occur like that we unable to go fishing because it destroys most of our boats which affects where we can get money…*” (FG,M) |
| 4 | Limited financial capital can restrict access to human capital, in the form of health services and education; *”… destroys most of our boats which affects where we can get money to take care of the children education, feeding at home and even save some for future emergency”…”I am here I have 6 children my only plan is to go fishing to take care of them but since the season didn’t favour me only one of my children is going to school. The rest of the 5 children are home and this is the problem we are going through in the coastal area”* (FG,M) |
| 5 | Limited financial capital may impact SWB by preventing individuals from undertaking social and cultural obligations to look after the family and the home; “*if my boat was not destroyed then I can take it fishing and get some catch to feed my family*” (FG,M) |
| 6 | Lower fish catches compared to before due to changing environmental conditions; “*nowadays the fishing season is not like it was in the past”* (CI,F) |
| 7 | Lower incomes compared to before, due to lower fish trading, can lower one’s perceived financial status; “*they do not buy from me as much as I would like. When it’s fishing season that is when they patronize my business [yet the fishing season is not as profitable as it once was]” (*CI,F) |
| 8 | Nevertheless, the town does have physical capital assets, including road networks and services in the town and nearby district capital; “*as we speak we are fortunate to have two banks in the town now which shows that this place is becoming a big town*” (FG,M)  Yet, this may cause negative impacts on SWB, including financial stress, if they perceive themselves to have “more-to-lose”; supported by the interaction between built-up landcover, storm exposure and financial stress (Model 3). |
| 9 | Damage to physical capital, including housing, can increase actors’ fears of climate hazards (natural perception), especially if past events have disturbed their peace or brought distress; “*So people who are relocating to the other side are not getting land so we are just living in fear because it can happen in the night*” (FG,M) |
| 10 | Coastal erosion has resulted in the community losing land over the years; “*the sea has taken over 60% of our land and still taking because the size of Town A is still reducing*” (FG,M) |
| 11 | Loss of land due to coastal erosion, and the need to relocate, can apply pressure upon reciprocal family/community relationships, with some respondents noting how they feel a burden if having to relocate with family members; “*I have five or six children and my sibling comes to live with me with their children too during this time [flooding], what will I do, how will I take care of all of us? So, when this happens then it becomes a burden*” (FG,F) |
| 12 | Past events and current worries can damage relationships and reduce actor’s attachment to place, linked to fears of displacement and relocation; “*People are displaced from their homes, you will not know where your neighbour is and you become disorganized. We do not know what to eat because the people who are selling the food are also trying to sort out their lives…[also] when the sea becomes violent [and] you are in somebody’s house you will not have peace”* (FG,F) |
| 13 | Loss of land due to coastal erosion can lower SWB. Drawing on the concept of “solastalgia”, individuals may experience distress if landscapes in which they are emotionally and culturally attached to are damaged or destroyed; “*as we speak the water bodies are taking the 30% [land] we have. We are in fear that in some years to we won’t have a place to settle*” (FG,M) |
| 14 | Loss of land to coastal erosion can increase the cost of the remaining land; “*back then when we had enough lands the cost of land isn’t that expensive and most of us could afford it*” (FG,M) |
| 15 | The eastern expansion of built infrastructure and employment opportunities from Accra and Tema into Ningo Prampram has increased the local population, land costs, conflicts and competition for services; “*the industrial areas are moving towards our district, they are expanding… they have a very huge population, they are the second biggest town in the district”* (DPO) |
| 16 | Increased land costs can generate perceived financial stress; “*due to the recent happenings the small lands are very scarce and expensive and all this makes us unhappy*” (FG,M) |
| 17 | Landscape characteristics, and the associated natural capital, are key sources for financial capital; however, these relationships are governed by relational processes associated with power and governance, and the “money-orientated” culture. For example, the sale of Songor Lagoon; “*government has taken a decision to give it to a private investor, and the investor pays back some revenue to national, to district assembly, whereby everybody benefits. So that is what the community is battling with now*” (DPO) |
| **No.** | **Description** |
| 17 (cont.) | Larger fishing vessels from other countries limiting the fish catches available to local artisanal fisherfolk; “*So the big boats are the ones spoiling our harvest because they also use certain types of chemicals which are not good for the sea*“ (FG,M)  Chiefs selling fishing rights to outsiders; “*in the past you could also take your tools and go and fish in the Paga, sell and buy things for yourself but now the chiefs are selling it so, we do not get anything*” (FG,F) |
| 18 | The autonomy to undertake desired livelihoods is restricted through the prevention of artisanal salt mining in Songor Lagoon; “*this was for us, it was for everyone in Ada. It was free, when you get there you could mine salt for free there and no one will take it from you or question you but that one is [now] for someone”…”In the past, you can go there with your basin collect the salt sell and use it to purchase things or pay off your debts but now it is no longer so”…” We are no longer happy so even if you are working you are disorganized*” (FG,F) |
| 19 | Traditional, intergenerational livelihood identities are prohibited by the sale of the Lagoon and the private police deployed to prevent access; “*You don’t have the right to take any salt out of the lagoon and this has brought about war between the town people and the individual the whole concession has been handed over to”* (FG,M) |
| 20 | The pride associated with fulfilling traditional identities may be limited if unable to continue salt mining, resulting in community-wide reductions in SWB; “*because the salt has been their livelihood for years. So, when they compare themselves to the other communities, they feel they are nothing*” (DPO) |
| 21 | Linking social capital, in the form of governmental support, is required to aid individuals who have had their traditional livelihoods removed adapt and diversify their income streams. However, this was not provided in the case of Ada West; “*So now that way of making money has been stopped and the disadvantage was that we did not put any alternative livelihood measures in place before the new arrangements, so that is what they are battling with*” (DPO) |
| 22 | Limited political influence in areas outside the district capital links to how decentralisation has resulted in district capital investment, yet the benefits and services, such as education and health (human capital), are not replicated in surrounding areas; “*There are a lot of inequalities...the district capital, is expected to have some basic facilities...the capital is developing and ideally it should be duplicating in other communities, but [it is not] because the assembly is [financially] handicapped”* (DPO) |
| 23 | Access to natural capital, which is influenced by the relational processes of governance, can result in conflict and the disruption of community relationships, as seen with the violence surrounding Songor Lagoon; “*The war [over the salt concession] is intense that we are fighting with guns and other weapons and this is affecting our peace in the community”* (FG,M) |
| 24 | Inequalities in physical capital may also influence how natural capital is accessed and used; for example, locals are unable to access salt during the wet season, yet outsiders with access to technology can maintain year-round access; “*We know of companies who mine nicely without having to carry it on your head but rather having some push trucks that helps bring it out. We town people uses manual way such that even after parking it outside and it rains it will wash it back into the lagoon*” (FG,M) |
| 25 | The inequality in natural capital access can result in inequalities in financial capital due to certain groups having access to more resources for trade during particular seasons; “*It is only the dry season you can get it so we can’t maintain it… gone were the days you can go and mine if you have the strength but now that they put it in the hand of Electrochem Company you can’t just go and mine unless you are an employee of the company*” (FG,M) |
| 26 | The restrictions on accessing Songor Lagoon have financial impacts, which can translate into subjective financial stress, with trade incomes lower than before, and also potentially more volatile due to limited livelihood options during the dry season where fish catches are lower; “*Like how we had money before now we don’t. Gone were the days when the school needs something, they go mining the salt to buy that*” (FG,M) |
| 27 | Climate hazards may influence social capital differently over time. Firstly, reciprocal relationships may strengthen during shocks and crises in order to overcome collective challenges; “*If there is an accident we all come together and help…if we all have to contribute one cedi we will do it****”*** (FG,F) |
| 28 | However, in other contexts, the unity and reciprocity between individuals is not as strong. For example, due to increasing competition and rising costs, there are weaker relationships when it comes to everyday trading and income generation; “*when we go to the market together and they buy mine but they do not buy hers then that is it, the relationship has been destroyed…instead of being happy for her you…carry hatred in your heart”* (FG,F) |
| 29 | Due to the threats of flooding, and the reduction in available fish catches, individuals (with sufficient adaptive capacity) are responding by adapting their livelihood practices; “*due to the disaster occurring the sea people have gone into salt mining*” (FG,M) |
| 30 | For example, individuals are altering their livelihood type by converting their farmland into salt mining sites; *“[the flood disaster is] making people turn their farmlands into mining sites*” (FG,M) |
| 31 | This land-use and livelihood shift has exacerbated soil salinity issues within the community; “*people just gather salt near farmland anyhow…through knowledge we got to know that the salt going through the grounds destroying things which makes crops flourish*” (FG,M) |

| **No.** | **Description** |
| --- | --- |
| 32 | The sensitivity of individuals’ farmland to salinity issues has been exacerbated by increasing populations, and the increased proximity of land used for salt mining/storage and crop production; “*Gone were the days the salt in the land doesn’t disturb the crops because people were not many but now due to the increment in population and the urge [to collect salt resources near others’ land]*” (FG,M) |
| 33 | The conversion of agricultural land to salt mining sites may also damage SWB by removing green spaces, and their associated aesthetic and health values; “*We are very happy about our environment because we are able to get fresh air and it’s natural”…”before when they plant crops here it was a beautiful sight*” (FG,M) |
| 34 | Fears of continued flood damage have resulted in investment taking place outside Anyamam in the district capital (Sege). Therefore, despite fishing businesses being located in the town, the financial capital received, and the consequent investment in infrastructure and dwellings, is extracted; “*they are using Anyamam as a business point and do their fishing and their everything, but in terms of assets they come and do it [in Sege]… they have their assets outside. He does not see that place as his permanent home*” (DPO) |
| 35 | The difference in investment creates proximate inequalities in infrastructure and services, which can enhance relative comparability and lower SWB; “*They do not renovate their house, not spend more money on it. So, they would rather invest it in the capital… if you ask him [with the investment resources] to construct toilets and stuff [in Anyamam] then he is not ready to do, because the floods can come and they will take it away*” (DPO) |


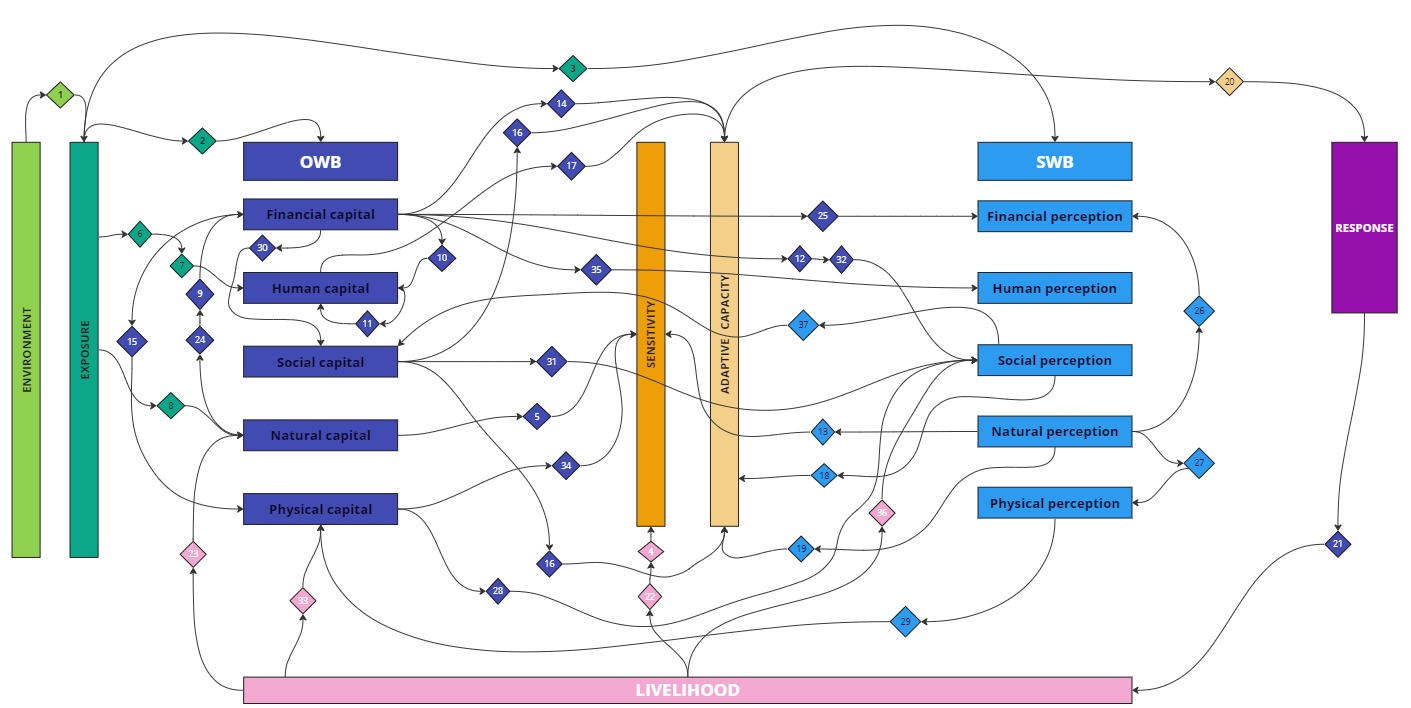


**Kedzi (Keta)**

| **Appendix 8:** Kedzi supporting evidence | |
| --- | --- |
| **No.** | **Description** |
| 1 | The community of Kedzi is exposed to multiple climate hazards, including; coastal erosion due to its location upon a thin spit between the ocean and Keta lagoon, uncertain rainfall and aquifer salinisation; “*if the dry season persists for a long time then it becomes salty…his borehole there is always salt in it*” (CI,M) |
| 2 | Across Volta Delta climate hazards can negatively impact OWB, illustrated by Models (1 & 2) showing drought to increase the odds of expenditure poverty and basic needs deprivation. |
| 3 | Across the study area, climate hazards such as salinisation, are associated with higher odds of unhappiness (Model 4); particularly regarding the domains of drinking water, environment and family/community interactions. |
| 4 | The livelihoods available in Kedzi influence environmental “sensitivity”; for example, households reliant upon fishing are sensitive to storm and flood hazards, “*the sea throw the fishing boats out of the sea leading to its destruction and some of the fishing boats were carried away and never found*” (FG,M). Similarly, farming livelihoods are sensitive to climate hazards in Kedzi; “***do your crops suffer during dry periods?*……***It affects me if the drought is for a while, so the groundwater goes down and sometimes my pipes are not able to get enough water*” (CI,M) |
| 5 | The natural capital and landscape in Kedzi may also increase the sensitivity of certain livelihoods to unfavourable environmental conditions; “*you see how sandy the place is, it is difficult as the crop will not survive*” (CI,M) |
| 6 | Hazards such as floods are also shown to negatively impact health, with increases in malaria and cholera during flood events; “*Most of them are used to open defecation…then it floods and it affects them…cholera increases during floods*” & “*Malaria is also very common during those times. During the rainy season, there is stagnant waters around your house and in other areas* a*nd you see mosquitos”* (CI,M) |
| 7 | Flooding can also disrupt schooling, limit children’s education, and restrict the transmission of knowledge to parents; “*during the rainy season, their homes, the whole compound is flooded…Some of the children just stay at home, and parents not give them pressure to come to school*” & *“educate the children so when they go home at least some of their parents, who did not have the opportunity to attend school, learn some of the basic things needed for good health”* (CI,M) |
| 8 | Climate hazards impact crop yields and fish catches (natural capital), “*storms affect the catch because if you capsize, all the fish is gone*” (CI,M) |
| 9 | Limited yield and catches inevitably impact the income available from trading produce; “*when the sea becomes rough, you cannot go fishing so you have to stay offshore [which limits how much you can offload and sell]*” (CI,M) |
| 10 | Limited income can feedback to restrict access to education; “*I was able to save a small amount, and I am doing my first degree at university*” (CI,M) |
| 11 | Limited education can feedback to further lower human capital in the form of health; “*I see no reason why I would allow my kids to sleep outside a net, when I know they will definitely get malaria…but [those without education] do not see that way, so I say education is lacking*” (CI,M) |
| 12 | Limited income may also threaten SWB by restricting individuals’ capacity to fulfil social obligations to provide for the family, particularly amongst male “breadwinners”’; “*I am with people around me, my family, with nothing in my pocket how will I support my family and the community. Someone from the community comes to you and asks for something and you have no money….how will you do, it is embarrassing*” (CI,M) |
| 13 | Past experiences of climate hazards may increase individuals’ sensitivity to further negative SWB impacts; “*if nothing is done [coastal defences] there’s the fear that in the next 2-5 years what we experience in the past will repeat again*” (FG,M) |
| 14 | Financial insecurity is a motivating factor behind adaptation, but initial financial capital is required to ensure sufficient adaptive capacity; *“[irrigation] requires money, because you cannot use your hands to water*” & ”*if you try farming, the land is not fertile enough, you have to invest beforehand*” (CI,M) |
| 15 | Financial capital is required to invest in the necessary physical capital to successfully adapt farming practices; “*first you pay electricity bills, and here the bills are a bit high, also have to buy the pipes to lay, the pumping machine, it can be a small amount of investment to be made*” (CI,M) |
| 16 | Social networks are used to access information and increase one’s capacity to adapt agricultural practices, including the installation of irrigation; “*What do we gain from neighbouring communities, I have a friend in the next town…I admire the work they are doing there. So, I come to him and said I also want to do some [irrigation] here. [He tells me to] buy this cow dung, this poultry waste and you spread it on the ground and then plant it. So, they encourage me and gave me guidance*” (CI,M) |
| 17 | A certain level of education is also required to ensure an individual has the adaptive capacity to implement new livelihood strategies; “*we are doing small farming here, there are things that are coming and without education you’ll still be hooked on the manual way of farming…there are things you need to use in your farm but because you have no idea, pest are worrying your crops and you don’t know what to do unless someone who is educated comes to see and tell you how to resolve the problem*” (FG,M) |
| 18 | Long-standing traditions and social perceptions regarding livelihood identity may also influence adaptive capacity. This idea is also linked to human/financial capital, as if the community has historically focused on fishing, then the skills required to adapt and undertake farming may not exist; “*the people in this community are not farmers, they are fisherfolk. You will be surprised the investment in farming, 100s cedis, the guy abandoned the work and left, so that money is down the drain*” (CI,M) |
| **No.** | **Description** |
| 19 | Attachment to land may increase one’s desire to protect the landscape and community, and therefore their adaptive capacity*; “The attachment or belonging that this is where I’m born and what I can do to make here a better place is the main reason why some of us are here…why we are still here protecting the little left for the future”* (FG,M) |
| 20 | The interviewee had sufficient OWB capitals to implement a “response” through irrigation; “*I farm as well did you see the garden opposite the clinic with irrigation, that is mine*” (CI,M) |
| 21 | The adaptation subsequently altered the individual’s livelihood. They were previously just a teacher, but now they possess a mixed livelihood as an irrigation farmer. |
| 22 | The change in livelihood and the installation of irrigation technology reduced the sensitivity to drought and uncertain seasonality; “*So as we are able to do irrigation [and grow okra year-round], and other farmers are waiting for rains*” (CI,M) |
| 23 | The adapted livelihood strategy increased access to more diverse natural capital, including cash crops; “*Onions and then currently Okra. It is a type of fruit that requires a lot of water*” (CI,M) |
| 24 | The importance of adaptation in improving financial wellbeing is illustrated by Model (1) with significantly lower odds of expenditure poverty in rural (high-EVI) areas when undertaking adaptive practice(s). The capacity for adaptive irrigation and higher-quality natural capital to improve financial OWB was illustrated by the individual’s market advantage over rain-fed farmers in the surrounding areas; “*the farmers are waiting for rains, so there is not as much of a supply, so when they hear we have okra here [due to irrigation] then they come rushing, and it is one of the fruits that people like using*” (CI,M) |
| 25 | Increased financial capital may result in less subjective financial stress, with the respondent noting how his increased salary has reduced financial worries as they can afford food and overcome basic challenges; “*now I am OK, because in life is simple. Once you have something to eat, you feed yourself and feed your family it is OK. If there are times when you do not have anything, when you get money mostly what worries is when you have nothing to eat, that is the biggest challenge*” (CI,M)  However, this relationship is a “relational” process, as the amount of financial capital required to fulfil an individual’s aspirations may shift over time; “*First salary came and I was so happy, now you feel OK but now the money does not mean as much. When I had nothing it was ok, but the more you earn the more responsibilities increase*” (CI,M) |
| 26 | The “relational context” in Kedzi, where approximately 80% of land had been lost to the ocean, including people’s dwellings and school infrastructure, may generate fears (natural perception), which could increase financial stress as residents are worried about losing any accumulated wealth; “*They worked hard and accumulated all those things and the rain just falls and washes it all* *away…If you are not careful the person will overthink things and die. They will be wondering where they will get the money…back after accumulating for a long time*” (FG,F) |
| 27 | Similarly, fears of future flooding due to repeated past hazards may have a cumulative effect and generate a sense of hopelessness regarding the material living standards available to them in Kedzi; “*you see as the sea keeps on removing us, people have still not relocated back. They don’t even visit here, they’ve migrated and are gone because the sea always disturb us a lot it just happen unexpectedly. You’ll just be there and it will flow into your room destroying your properties even it pulls down houses*” (FG,M) |
| 28 | Damage to assets can also lower one’s status in the community, which can negatively impact their self-evaluated SWB; “*when all your assets get destroyed through those hazards it reduces the recognition and prestige you might have in the community*” (FG,M) |
| 29 | Fears of future hazards and destruction may ultimately feedback to disincentivize investment in improving the physical capital of the town; “*we are currently in fear of amassing assets then later these disaster strikes again…Even though people would like to do things to beautify the community but due to the fear of disaster they don’t engage in it*” (FG,M) |
| 30 | The ability to increase financial capital through adaptive irrigation may strengthen social capital, with additional funds able to reinforce reciprocal family relationships; “*I have my mum at home, she’s 60 something and I have to support her to survive*” (CI,M) |
| 31 | Fulfilling reciprocal family relationships can also be assumed to improve SWB, with Ghanaian studies highlighting the importance of fulfilling traditional obligations in achieving a “good life”; “*I always say family is the most important one. Also, when something happen to me now my families are the first people to come and help before the society will even hear it*” (FG,M) |
| 32 | Improved financial wellbeing may also improve one’s social status, resulting in increased happiness. This relationship was illustrated by the assemblyman noting his admiration for a community member who had accumulated financial capital and assets; “*No… money if not in your pocket you are nobody….[when describing the person who is “doing the best”] He has a private car for himself, three boats working for him, he deserved to be applauded. He is serious with his business*” (CI,M)  This effect of financial capital upon social status is “relational”, with respondents noting how the value attached to financial capital has shifted over time, and is especially prominent amongst the youth of today; “*the attitude of the youth about work, all they need right now is money in their pocket*” (CI,M) |

| **No.** | **Description** |
| --- | --- |
| 33 | The incorporation of irrigation farming livelihoods, and the subsequent market advantage, reduced the importance of having high quality physical capital in the form of road networks as people visited the individual to buy their produce, rather than having to commute to markets; ”*Yes people come to buy, people in the community and people from other sites if they need it then they come. I do not travel to the market*” (CI,M) |
| 34 | Due to buyers visiting the farm itself due to the limited supply of high-moisture crops during dry periods, the individual’s financial sensitivity to physical remoteness from key markets was reduced. |
| 35 | Access to increased trade incomes also improved the individual’s autonomy (human perception), as the higher wage meant that they were no longer dependent upon others; “*I have the opportunity to attend school, I have not reached [where I want to yet], but at least I do not depend on anyone to survive*” (CI,M) |
| 36 | Overall, the adaptive process and the change in livelihood fulfilled the social expectation of “working hard” in the community. Working diligently to progress one’s business and support the family is a desired trait, which if fulfilled can boost individuals’ pride and SWB; ”*what I am doing, I am going to work, I wake up at 3am, then 6am take a shower before work, then come back and do it again*” (CI,M) |
| 37 | However, this social perception may weaken tangible social relationships, as the interviewee noted his frustrations with others who were given similar opportunities to undertake irrigation farming, but did not put the required effort in; “*two guys who were with us yesterday. Someone invested, laid the pipes, it is quite expensive… these two guys cannot wake up to weed, they do not go to work, they are in the house, maybe they go fishing and then they come back… the way they took care of grasses, weeds took over crops, the man [gave up] because they are making loss*” (CI,M) & “*what he’s doing [the person doing the worst (0/10)] is not yielding any profit, what he is doing there is nothing to show*” (CI,M) |

**References**

Addison, T., Hulme, D., & Kanbur, R. (2008). Poverty dynamics: measurement and understanding from an interdisciplinary perspective. Brooks World Poverty Institute Working Paper No.19.

Adger, W. N., Kelly, P. M., Winkels, A., Huy, L. Q., & Locke, C. (2002). Migration, remittances, livelihood trajectories, and social resilience. *AMBIO: A Journal of the Human Environment*, *31*(4), 358-366, <https://doi.org/10.1579/0044-7447-31.4.358>

Agarwala, M., Atkinson, G., Fry, B. P., Homewood, K., Mourato, S., Rowcliffe, J. M., Wallace, G., & Milner-Gulland, E. (2014). Assessing the relationship between human well-being and ecosystem services: a review of frameworks. *Conservation and Society*, *12*(4), 437-449. https://doi.org/10.4103/0972-4923.155592

Aguilar, G., & Sumner, A. (2020). Who are the world’s poor? A new profile of global multidimensional poverty. *World Development*, *126*(104716), 1–15. https://doi.org/10.1016/j.worlddev.2019.104716

Alam, E., & Mallick, B. (2022). Climate change perceptions, impacts and adaptation practices of fishers in southeast Bangladesh coast. *International Journal of Climate Change Strategies and Management*, *14*(2), 191–211. https://doi.org/10.1108/ijccsm-02-2021-0019

Alkire, S., & Foster, J. (2011). Counting and multidimensional poverty measurement. *Journal of Public Economics*, *95*(7–8), 476–487. https://doi.org/10.1016/j.jpubeco.2010.11.006

Alkire, S., & Santos, M. E. (2010). Acute multidimensional poverty: A new index for developing countries. https://ophi.org.uk/wp-38/; last accessed 2nd April 2023.

Arndt, C. & Tarp, F. (2016). *Measuring poverty and wellbeing in developing countries*. Oxford University Press, Oxford. https://doi.org/10.1093/acprof:oso/9780198744801.001.0001

Batana, Y. M., & Cockburn, J. (2018). Do demographics matter for African child poverty? *World Bank Policy Research Working Paper* (8426), <https://doi.org/10.1596/1813-9450-8426>

Begum, A., Lempert, R., Ali, E., Bernauer, T., Cramer, W., Cui, X., Mach, K., Nagy, G., Steseth, N.C., Sukumar, R. & Wester, P. (2022). In Pörtner H-O., Roberts, D.C., Tignor, M., Poloczanska, E.S., Mintenbeck, K., Alegría, A., Craig, M., Langsdorf, S., Löschke, S., Möller, V., Okem, A. & Rama, B (eds) *Climate Change 2022: Impacts, Adaptation, and Vulnerability. Contribution of Working Group II to the Sixth Assessment Report of the Intergovernmental Panel on Climate Change* (pp. 121-196). Cambridge University Press, Cambridge, https://doi.org/10.1017/9781009325844.003

Berchoux, T., & Hutton, C. W. (2019). Spatial associations between household and community livelihood capitals in rural territories: An example from the Mahanadi Delta, India. *Applied Geography*, 103, 98-111. https://doi.org/10.1016/j.apgeog.2019.01.002

Bouma, J., Bulte, E., & Van Soest, D. (2008). Trust and cooperation: Social capital and community resource management. *Journal of environmental economics and management, 56*(2), 155-166, https://doi.org/10.1016/j.jeem.2008.03.004

Brüggen, E. C., Hogreve, J., Holmlund, M., Kabadayi, S., & Löfgren, M. (2017). Financial well-being: A conceptualization and research agenda. *Journal of Business Research*, *79*, 228–237. https://doi.org/10.1016/j.jbusres.2017.03.013

Cooper, P. (2013). Socio-ecological accounting: DPSWR, a modified DPSIR framework, and its application to marine ecosystems. *Ecological economics*, *94*, 106-115. https://doi.org/10.1016/j.ecolecon.2013.07.010

Copestake, J. (2008). Wellbeing in international development: What's new?. Journal of International Development: *The Journal of the Development Studies Association, 20*(5), 577–597. https://doi.org/10.1002/jid.1431

Coulthard, S. (2011). More than just access to fish: the pros and cons of fisher participation in a customary marine tenure (Padu) system under pressure. *Marine Policy*, *35*(3), 405-412. https://doi.org/10.1016/j.marpol.2010.11.006

Cummins, R. A., Eckersley, R., Pallant, J., Van Vugt, J., & Misajon, R. (2003). Developing a national index of subjective wellbeing: The Australian Unity Wellbeing Index. *Social indicators research, 64*(2), 159-190, https://doi.org/10.1023/A:1024704320683

Dasgupta, A., & Baschieri, A. (2010). Vulnerability to climate change in rural Ghana: Mainstreaming climate change in poverty‐reduction strategies. *Journal of International Development*, *22*(6), 803-820. https://doi.org/10.1002/jid.1666

Davis, E. P. (2014). A review of the economic theories of poverty. National Institute of Economic and Social Research Discussion Paper No. 435.

Daw, T., Brown, K., Rosendo, S., & Pomeroy, R. (2011). Applying the ecosystem services concept to poverty alleviation: the need to disaggregate human well-being. *Environmental Conservation*, *38*(4), 370-379. https://doi.org/10.1017/S0376892911000506

De Schutter, O. (2021). A human rights-based approach to measuring poverty. In Davis, M., Kjaerum, M. & Lyons, A. (eds) *Research Handbook on Human Rights and Poverty* (pp-2-20), Edward Elgar Publishing, Cheltenham. https://doi.org/10.4337/9781788977517.00010

Diener, E. & Suh, E. (1997). Measuring quality of life: Economic, social, and subjective indicators. *Social indicators research*, *40*(1): 189–216. https://doi.org/10.1023/A:1006859511756

Dolan, P., & White, M. P. (2007). How Can Measures of Subjective Well-Being Be Used to Inform Public Policy? *Perspectives on Psychological Science : A Journal of the Association for Psychological Science*, *2*(1), 71–85. https://doi.org/10.1111/j.1745-6916.2007.00030.x

Estoque, R. C., Ishtiaque, A., Parajuli, J., Athukorala, D., Rabby, Y. W., & Ooba, M. (2023). Has the IPCC’s revised vulnerability concept been well adopted? *Ambio*, *52*(2), 376-389. https://doi.org/10.1007/s13280-022-01806-z

Fagerholm, N., Oteros-Rozas, E., Raymond, C. M., Torralba, M., Moreno, G., & Plieninger, T. (2016). Assessing linkages between ecosystem services, land-use and well-being in an agroforestry landscape using public participation GIS. *Applied Geography*, 74, 30-46. https://doi.org/10.1016/j.apgeog.2016.06.007

Fisher, J. A., Patenaude, G., Meir, P., Nightingale, A. J., Rounsevell, M. D., Williams, M., & Woodhouse, I. H. (2013). Strengthening conceptual foundations: analysing frameworks for ecosystem services and poverty alleviation research. *Global environmental change*, *23*(5), 1098-1111. https://doi.org/10.1016/j.gloenvcha.2013.04.002

Flik, R. J., & van Praag, B. M. S. (1991). Subjective poverty line definitions. *De Economist*, *139*(3), 311–330. https://doi.org/10.1007/bf01423569

Gari, S. R., Newton, A., & Icely, J. D. (2015). A review of the application and evolution of the DPSIR framework with an emphasis on coastal social-ecological systems. *Ocean & Coastal Management*, *103*, 63-77. https://doi.org/10.1016/j.ocecoaman.2014.11.013

Ghai, D. (1978). Basic needs and its critics. *The IDS Bulletin, 9*(4), 16–18. https://doi.org/10.1111/j.1759-5436.1978.mp9004004.x

González, P., Sehnbruch, K., Apablaza, M., Méndez Pineda, R., & Arriagada, V. (2021). A Multidimensional Approach to Measuring Quality of Employment (QoE) Deprivation in Six Central American Countries. *Social Indicators Research*, *158*(1), 107–141. https://doi.org/10.1007/s11205-021-02648-0

Gough, I. & McGregor, J. A. (2007). *Wellbeing in developing countries: from theory to research.* Cambridge University Press, Cambridge.

Gross-Camp, N. (2017). Tanzania’s community forests: their impact on human well-being and persistence in spite of the lack of benefit. *Ecology and Society, 22*(1). https://doi.org/10.5751/ES-09124-220137

GSS. (2018). Poverty trends in Ghana 2005-2017, a report produced from the Ghana Living Standards Survey Round 7 (GLSS7). https://www2.statsghana.gov.gh/docfiles/publications/ GLSS7/Poverty%20Profile%20Report_2005%20-%202017.pdf, last accessed 4^th^ October 2024.

GSS. (2020). *Multidimensional Poverty - Ghana*. <https://ophi.org.uk/wp-content/uploads/Ghana_MPI_report_2020.pdf>; last accessed 5^th^ August 2023.

Hjelm, L., Ferrone, L., Handa, S. & Chzhen, Y. (2016). Comparing approaches to the measurement of multidimensional child poverty, a UNICEF Office of Research Innocenti Working Paper 2016/29.

IPCC. (2024). *IPCC Glossary*. <https://apps.ipcc.ch/glossary/>, last accessed 7^th^ March 2024

Jehan, S., & Umana, A. (2003). The environment-poverty nexus. *Development Policy Journal*, *3*(20), 53-70.

Kay, A. C., & Jost, J. T. (2003). Complementary justice: effects of" poor but happy" and" poor but honest" stereotype exemplars on system justification and implicit activation of the justice motive. *Journal of personality and social psychology*, *85*(5), 823, <https://doi.org/10.1037/0022-3514.85.5.823>

Kelble, C. R., Loomis, D. K., Lovelace, S., Nuttle, W. K., Ortner, P. B., Fletcher, P., Cook, G. S., Lorenz, J. J., & Boyer, J. N. (2013). The EBM-DPSER conceptual model: integrating ecosystem services into the DPSIR framework. *Plos one*, *8*(8), e70766. https://doi.org/10.1371/journal.pone.0070766

Kozma, A., Stone, S., & Stones, M. (2000). Stability in components and predictors of subjective well-being (SWB): Implications for SWB structure. In Diener, E and Rahtz, D (Editors) *Advances in quality of life theory and research* (pp. 13-30). Springer. https://doi.org/10.1007/978-94-011-4291-5_2

Laderchi, C. R., Saith, R., & Stewart, F. (2003). Does it matter that we do not agree on the definition of poverty? A comparison of four approaches. *Oxford development studies*, *31*(3), 243-274, <https://doi.org/10.1080/1360081032000111698>

Leach, M., Mearns, R., & Scoones, I. (1999). Environmental entitlements: dynamics and institutions in community-based natural resource management. *World Development*, *27*(2), 225-247. https://doi.org/10.1016/S0305-750X(98)00141-7

Lent, R. W. (2004). Toward a unifying theoretical and practical perspective on well-being and psychosocial adjustment. *Journal of Counseling Psychology*, *51*(4), 482-509. https://doi.org/10.1037/0022-0167.51.4.482

Mahmood, T., Yu, X., & Klasen, S. (2018). Do the Poor Really Feel Poor? Comparing Objective Poverty with Subjective Poverty in Pakistan. *Social Indicators Research*, *142*(2), 543–580. https://doi.org/10.1007/s11205-018-1921-4

Marks, G. N. (2007). Income poverty, subjective poverty and financial stress, an Australian Government Social Policy Research Paper no. 29.

MEA. (2005). *Ecosystems and human well-*being (Vol. 5). Island press Washington, DC.

Nuijten, E. (2011). Combining research styles of the natural and social sciences in agricultural research. *NJAS: Wageningen Journal of Life Sciences*, *57*(3-4), 197-205. https://doi.org/10.1016/j.njas.2010.10.003

Nunan, F. (2015). *Understanding poverty and the environment: analytical frameworks and approaches*. Routledge, Oxon. https://doi.org/10.4324/9781315886701

OECD. (2011). *What are equivalence scales?* https://www.oecd.org/els/soc/OECD-Note-EquivalenceScales.pdf, last accessed 5^th^ July 2023.

Petesch, P., & Narayan, D. (2002). *Voices of the poor: from many lands.* Oxford University Press and the World Bank; Washington. https://hdl.handle.net/10986/14053

Rekolainen, S., Kämäri, J., Hiltunen, M., & Saloranta, T. M. (2003). A conceptual framework for identifying the need and role of models in the implementation of the Water Framework Directive. *International Journal of River Basin Management*, *1*(4), 347-352. https://doi.org/10.1080/15715124.2003.9635217

Reyes-García, V., Babigumira, R., Pyhälä, A., Wunder, S., Zorondo-Rodríguez, F., & Angelsen, A. (2015). Subjective Wellbeing and Income: Empirical Patterns in the Rural Developing World. *Journal of Happiness Studies*, *17*(2), 773–791. https://doi.org/10.1007/s10902-014-9608-2

Rojas, M. (2006). Life satisfaction and satisfaction in domains of life: is it a simple relationship? *Journal of Happiness Studies*, *7*(4), 467–497. https://doi.org/10.1007/s10902-006-9009-2

Rojas, M. (2011). The ‘Measurement of economic performance and social progress’ report and quality of life: moving forward. *Social Indicators Research, 102*(1), 169–180. https://doi.org/10.1007/s11205-010-9737-x

Santos, M. E., & Villatoro, P. (2018). A Multidimensional Poverty Index for Latin America. *Review of Income and Wealth*, *64*(1), 52–82. https://doi.org/10.1111/roiw.12275

Schwarz, N., & Strack, F. (1999). Reports of subjective well-being: Judgmental processes and their methodological implications. In D. Kahneman, E. Diener, & N. Schwarz (Editors) *Well-being: The foundations of hedonic psychology* (pp. 61–84). Russell Sage Foundation.

Scoones, I. (1998). Sustainable rural livelihoods: a framework for analysis, an Institute of Development Studies (IDS) working paper no. 72.

Scott, L. (2006). Chronic poverty and the environment: A vulnerability perspective. *Chronic Poverty Research Centre Working Paper*(62), <https://doi.org/10.2139/ssrn.1753643>

Sen, A. (1983). Poor, Relatively Speaking. *Oxford Economic Papers*, *35*(2), 153–169. https://doi.org/10.1093/oxfordjournals.oep.a041587

Sen, A. (1999). *Commodities and capabilities.* Oxford University Press, Oxford.

Small, N., Munday, M., & Durance, I. (2017). The challenge of valuing ecosystem services that have no material benefits. *Global environmental change*, *44*, 57-67. https://doi.org/10.1016/j.gloenvcha.2017.03.005

Streeten, P. (1984). Basic needs: some unsettled questions. *World Development*, *12*(9), 973–978. https://doi.org/10.1016/0305-750X(84)90054-8

Tangney, P. (2020). Understanding climate change as risk: a review of IPCC guidance for decision-making. *Journal of Risk Research*, *23*(11), 1424-1439. https://doi.org/10.1080/13669877.2019.1673801

TEEB. (2022). *Approach*. http://teebweb.org/about/approach/, last accessed 11^th^ October 2024.

Thomas, K., Hardy, R. D., Lazrus, H., Mendez, M., Orlove, B., Rivera‐Collazo, I., Roberts, J. T., Rockman, M., Warner, B. P., & Winthrop, R. (2019). Explaining differential vulnerability to climate change: A social science review. *Wiley Interdisciplinary Reviews: Climate Change*, *10*(2), e565, <https://doi.org/10.1002/wcc.565>

Vyas, S., & Kumaranayake, L. (2006). Constructing socio-economic status indices: how to use principal components analysis. *Health Policy and Planning*, *21*(6), 459–468. https://doi.org/10.1093/heapol/czl029

White, S. C. (2010). Analysing wellbeing: a framework for development practice. *Development in Practice*, *20*(2), 158–172. https://doi.org/10.1080/09614520903564199

1. Endowments refer to households’ rights to resources/services (i.e. land ownership) (Leach, 1999) [↑](#footnote-ref-1)
2. Entitlements are the “set of utilities derived from environmental goods/services over which social actors have legitimate…command and which are instrumental in achieving well-being” (i.e. access to market value for resources) (Leach et al., 1999; p.233). [↑](#footnote-ref-2)
